# Synergistic involvement of the NZF domains of the LUBAC accessory subunits HOIL-1L and SHARPIN in the regulation of LUBAC function

**DOI:** 10.1038/s41419-024-07199-z

**Published:** 2024-11-11

**Authors:** Yusuke Toda, Hiroaki Fujita, Koshiki Mino, Takuto Koyama, Seiji Matsuoka, Toshie Kaizuka, Mari Agawa, Shigeyuki Matsumoto, Akiko Idei, Momoko Nishikori, Yasushi Okuno, Hiroyuki Osada, Minoru Yoshida, Akifumi Takaori-Kondo, Kazuhiro Iwai

**Affiliations:** 1 Department of Molecular and Cellular Physiology, Graduate School of Medicine, Kyoto University, Sakyo-ku, Kyoto, Kyoto, 606-8501 Japan; 2 Department of Hematology and Oncology, Graduate School of Medicine, Kyoto University, Sakyo-ku, Kyoto, Kyoto, 606-8507 Japan; 3 Drug Discovery Seed Compounds Exploratory Unit, RIKEN Center for Sustainable Resource Science, Wako, Saitama 351-0198 Japan; 4 Department of Biomedical Data Intelligence, Graduate School of Medicine, Kyoto University, Sakyo-ku, Kyoto, Kyoto, 606-8507 Japan; 5 Human Health Sciences, Graduate School of Medicine, Kyoto University, Sakyo-ku, Kyoto, Kyoto, 606-8501 Japan; 6 Chemical Resource Development Research Unit, RIKEN Center for Sustainable Resource Science, Wako, Saitama 351-0198, Japan; Department of Pharmaceutical Sciences, University of Shizuoka, Shizuoka, Shizuoka, 422-8526 Japan

**Keywords:** Cell signalling, Apoptosis

## Abstract

The linear ubiquitin chain assembly complex (LUBAC) plays crucial roles in NF-κB signaling and protection against cell death by generating linear ubiquitin chains. Its accessory subunits, HOIL-1L and SHARPIN, regulate LUBAC function by binding to ubiquitin chains via their Npl4 zinc finger (NZF) domains. However, the synergistic effects of the two NZF domains on LUBAC function remain unclear. Here, we demonstrate that the ubiquitin-binding activity of the two NZF domains cooperatively regulates LUBAC functions. Simultaneous loss of the ubiquitin-binding activity of the NZF domains profoundly impaired both NF-κB activation and cell death protection functions. HOIL-1L NZF robustly binds to linear ubiquitin chains, whereas SHARPIN NZF binds to Lys(K)63-linked ubiquitin chains in addition to linear chains. Binding of both NZF domains to linear ubiquitin chains regulated NF-κB signaling, whereas SHARPIN NZF predominantly regulated the cell death protection function independently of the ubiquitin chain type, K63-linked or linear ubiquitin. However, concomitant loss of linear ubiquitin binding by HOIL-1L NZF drastically impaired cell death protection. A screen of compounds capable of inhibiting binding between HOIL-1L NZF and linear ubiquitin chains identified a small compound that inhibited SHARPIN NZF as well as HOIL-1L NZF binding to linear ubiquitin chains, supporting the synergistic effect of the two NZF domains on cell death protection and suggesting a potential therapeutic strategy for targeting increased LUBAC activity in diseases such as cancer.

## Introduction

The ubiquitin system plays a critical role in regulating diverse physiological processes through the conjugation of ubiquitin, mostly in the form of ubiquitin chains [1–4]. Different ubiquitin chains are formed via one of seven lysine residues, and the type of ubiquitin chain determines the functions of the modified proteins [5, 6]. For instance, Lys(K)48-linked ubiquitin chains target proteins for proteasomal degradation, whereas K63-linked ubiquitin chains are involved in signal transduction processes [2, 3, 5, 6]. We discovered a novel linear ubiquitin chain linked via the amino-terminal Met-1 of ubiquitin and assembled by the linear ubiquitin chain assembly complex (LUBAC), a ubiquitin E3 ligase complex that specifically generates linear chains [7]. LUBAC functions in canonical NF-κB activation and protection against programmed cell death [8–16].

Linear ubiquitination plays a critical role in tumor necrosis factor (TNF)-α signaling. Upon binding to TNF-α, the TNF receptor I (TNFR1) initiates the formation of TNFR1 signaling complex (complex I), which consists of receptor interacting Ser/Thr-protein kinase 1 (RIPK1), TNFR-associated death domain (TRADD), TNF receptor-associated factor 2 (TRAF2), and cellular inhibitor of apoptosis proteins 1 and 2 (cIAP1/2) [10, 17]. Subsequently, LUBAC is recruited to complex I through recognition of K63-linked ubiquitin chains attached to the components of the complex mainly by cIAP1/2 [8, 9, 18]. LUBAC then conjugates linear ubiquitin chains to NF-κB essential modulator, the regulatory protein of the IKK complex, to activate NF-κB [8, 9, 18–20]. LUBAC also modifies components of complex I including TNFR1 and RIPK1, which leads to protection against cell death by inhibiting the formation of a cytosolic complex (complex II) containing RIPK1, RIPK3, TRADD, FAS-associated death domain protein, and caspase-8 [10, 17, 21].

LUBAC is composed of three subunits: a catalytic HOIP, and two accessory HOIL-1L and SHARPIN subunits [11]. Both accessory subunits are involved in stabilizing LUBAC by binding to HOIP and each other via their ubiquitin-like and LUBAC-tethering motif domains, respectively, and they regulate LUBAC function by binding to ubiquitin chains through their Npl4 zinc finger (NZF) domains [12, 13, 22–24]. Although both NZFs bind to linear ubiquitin chains, HOIL-1L NZF exhibits robust binding affinity for linear chains [23, 25], whereas SHARPIN NZF binds to K63-linked ubiquitin chains in addition to linear chains, with which it interacts less efficiently than HOIL-1L NZF [13, 23, 24]. It has been suggested that HOIL-1L NZF contributes to NF-κB activation and SHARPIN NZF is involved in both NF-κB activation and cell death protection [12, 13, 23–25]; however, their cooperative effect on LUBAC function remains to be elucidated.

In this study, we show that the binding of these NZF domains to ubiquitin chains cooperatively regulates LUBAC function. The binding of both NZFs to linear ubiquitin chains played a predominant role in NF-κB activation. By contrast, SHARPIN NZF primarily contributed to cell death protection, regardless of the ubiquitin chain type, and synergistic loss of ubiquitin binding of both NZFs drastically impaired cell death protection.

## Results

### The ubiquitin-binding activity of HOIL-1L NZF preferentially contributes to NF-κB activation

**Fig. 1:**
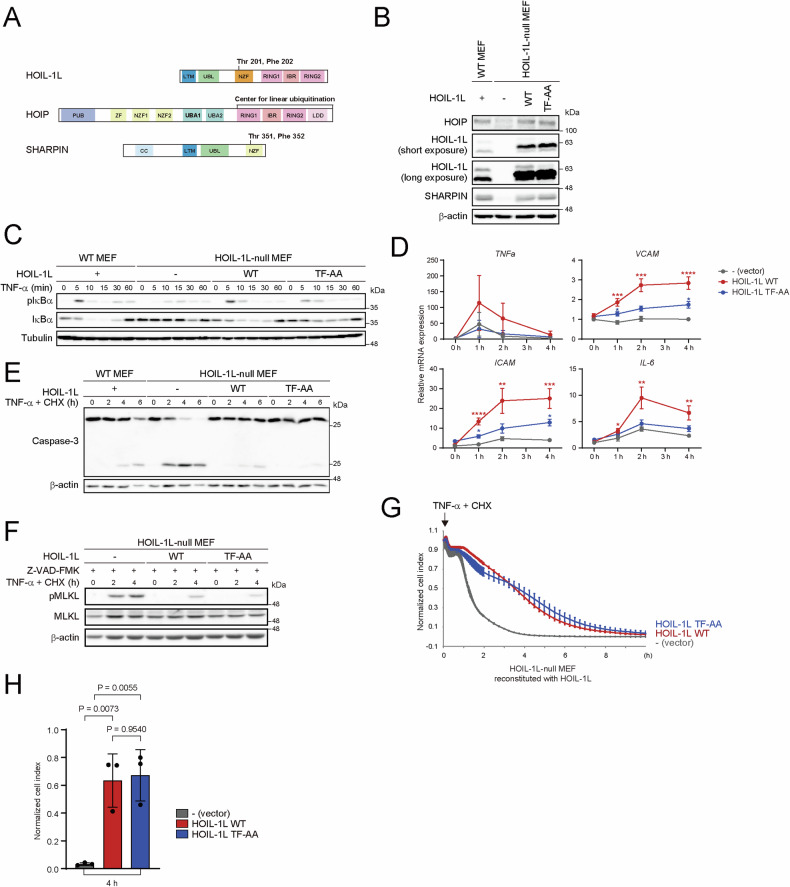
The ubiquitin-binding activity of HOIL-1L NZF preferentially contributes to NF-κB activation. **A** Schematic illustration of the domains of LUBAC subunits. **B** Immunoblot analyses of lysates of WT MEFs and HOIL-1L-null MEFs stably reconstituted with the indicated proteins. Data are representative of three independent experiments. **C**, **E** WT MEFs and HOIL-1L-null MEFs stably reconstituted with the indicated proteins were stimulated with TNF-α (1 ng/mL) (**C**) or TNF-α (2.5 ng/mL) plus CHX (20 μg/mL) (**E**) for the indicated times, and analyzed by immunoblotting with the indicated antibodies. Data are representative of three independent experiments. **D** HOIL-1L-null MEFs stably reconstituted with the indicated proteins were stimulated with TNF-α (1 ng/mL), and expression of NF-κB target genes was measured by quantitative PCR. All gene expression levels are normalized against the corresponding levels of *ACTB* and expressed as fold changes relative to cells with an empty vector at 0 h. Data are shown as the mean ± s.d. of three independent experiments. *P* values were calculated by a one-way ANOVA with Tukey’s post hoc test. *P < 0.05, **P < 0.01, ***P < 0.001 and ****P < 0.0001. **F** HOIL-1L-null MEFs stably reconstituted with the indicated proteins were stimulated with TNF-α (2.5 ng/mL) and CHX (20 μg/mL) for the indicated times after pretreatment with Z-VAD-FMK (10 μM) for 1 h, and analyzed by immunoblotting with the indicated antibodies. Data are representative of three independent experiments. **G, H** HOIL-1L-null MEFs stably reconstituted with the indicated proteins were stimulated with TNF-α (2.5 ng/mL) and CHX (20 μg/mL), and cell viability was continuously measured using the xCELLigence system. A representative image (**G**) and statistical analyses of the normalized cell index at 4 h (**H**). Data are shown as the mean ± s.d. of three independent experiments. *P* values were calculated by a one-way ANOVA with Tukey’s post hoc test. Uncropped western blots are available for this figure in the Supplementary Material.

The Thr and Phe amino acid residues of NZF domains are critical for the interaction with ubiquitin chains [26, 27]. The ubiquitin-binding activity of the NZFs of HOIL-1L and SHARPIN was investigated by generating TF-AA mutants in which Thr and Phe of the NZF domains were substituted with Ala in HOIL-1L (HOIL-1L T201A/F202A: HOIL-1L TF-AA) and SHARPIN (SHARPIN T351A/F352A: SHARPIN TF-AA) (Fig. 1A, Supplementary Fig. 1A). To determine whether both TF-AA mutants lose ubiquitin-binding activity, purified mutant proteins were subjected to in vitro ubiquitin-binding assays, which confirmed that both TF-AA mutants could not bind to the corresponding ubiquitin chains (Supplementary Fig. 1B, C). When co-expressed with HOIP, the HOIL-1L TF-AA mutant could not activate NF-κB to a comparable extent as the HOIL-1L T201A/R208A mutant (HOIL-1L TR-AA), which lacks linear ubiquitin-binding activity and fails to activate NF-κB when co-expressed with HOIP [23] (Supplementary Fig. 1D). We then retrovirally introduced wild-type (WT) or TF-AA mutant HOIL-1L into mouse embryonic fibroblasts (MEFs) lacking HOIL-1L. The amount of HOIP, which reflects the amount of LUBAC, in MEFs expressing HOIL-1L WT or TF-AA was comparable with that in WT MEFs (Fig. 1B). Loss of HOIL-1L attenuated TNF-α-mediated phosphorylation and degradation of IκBα, which are hallmarks of NF-κB activation, and HOIL-1L WT restored this effect almost completely. However, the TF-AA mutant failed to restore NF-κB activation and failed to induce expression of NF-κB target genes effectively (Fig. 1C, D). Loss of HOIL-1L also sensitizes cells to TNF-α-induced cell death by reducing the amount of LUBAC [22]. Unlike the effect on NF-κB activation, evaluation of apoptosis and necroptosis upon treatment with TNF-α and cycloheximide (CHX) showed that not only HOIL-1L WT, but also the TF-AA mutant, significantly attenuated TNF-α-induced cell death (Fig. 1E–H). Upon treatment with TNF-α and SM-164, both HOIL-1L WT and the TF-AA mutant also suppressed cell death, although HOIL-1L TF-AA was less effective than HOIL-1L WT (Supplementary Fig. 2A–C).

### The NZF domains in HOIL-1L and SHARPIN cooperatively regulate NF-κB activation mainly by binding to linear ubiquitin chains

**Fig. 2:**
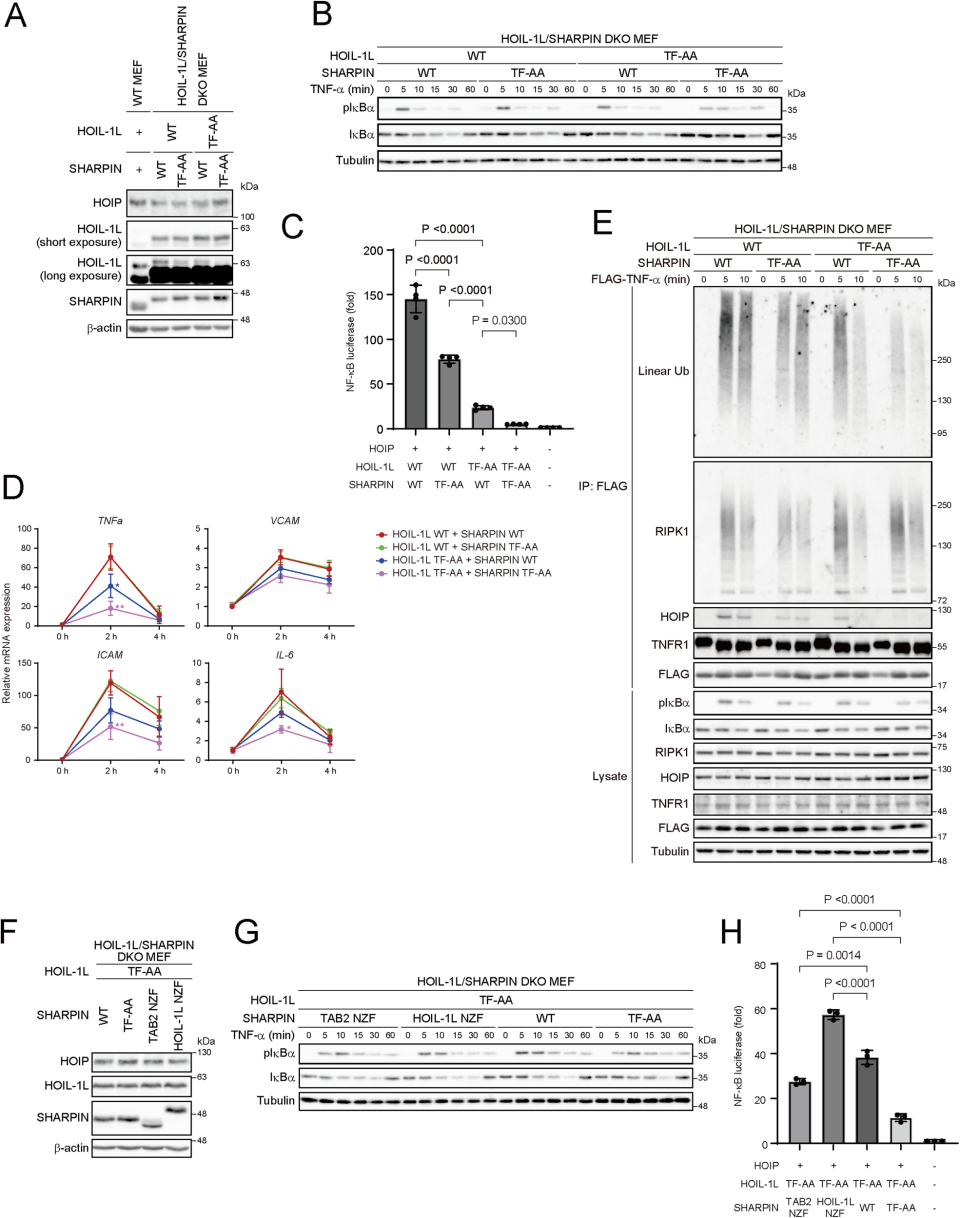
The NZF domains in HOIL-1L and SHARPIN cooperatively regulate NF-κB activation mainly by binding to linear ubiquitin chains. **A, F** Immunoblot analyses of lysates of WT MEFs and HOIL-1L/SHARPIN DKO MEFs stably reconstituted with the indicated proteins. Data are representative of three independent experiments. **B, G** HOIL-1L/SHARPIN DKO MEFs stably reconstituted with the indicated proteins were stimulated with TNF-α (5 ng/mL) for the indicated times and analyzed by immunoblotting with the indicated antibodies. Data are representative of three independent experiments. **C, H** NF-κB activation in HEK293T cells transiently transfected with the indicated combinations of HOIP, HOIL-1L, and SHARPIN was measured using a luciferase assay. Data are shown as the mean ± s.d. of four (**C**) or three (**H**) independent experiments. *P* values were calculated by one-way ANOVA with Tukey’s post hoc test. **D** HOIL-1L/SHARPIN DKO MEFs stably reconstituted with the indicated proteins were stimulated with TNF-α (5 ng/mL), and expression of NF-κB target genes was measured by quantitative PCR. All gene expression levels are normalized against the corresponding levels of *ACTB* and expressed as fold changes relative to cells with HOIL-1L WT and SHARPIN WT at 0 h. Data are shown as the mean ± s.d. of three independent experiments. *P* values were calculated by a one-way ANOVA with Tukey’s post hoc test. *P < 0.05 and **P < 0.01. **E** HOIL-1L/SHARPIN DKO MEFs stably reconstituted with the indicated proteins were stimulated with FLAG-tagged TNF-α (1 μg/mL) for the indicated times. Lysates were immunoprecipitated with anti-FLAG antibody and immunoblotted as indicated. The experiments were repeated three times, independently, with similar results. Uncropped western blots are available for this figure in the Supplementary Material.

We next investigated whether both NZF domains in HOIL-1L and SHARPIN exert synergic effects on two LUBAC functions: NF-κB activation and protection from cell death. To this end, we introduced the WT or TF-AA mutants of HOIL-1L and SHARPIN into MEFs lacking HOIL-1L and SHARPIN (double knockout (DKO) MEFs) (Supplementary Fig. 3), as shown in Fig. 2A. We first evaluated their effects on NF-κB activation. Upon stimulation with TNF-α, phosphorylation and degradation of IκBα were attenuated in DKO MEFs carrying the TF-AA mutant of HOIL-1L or SHARPIN, although HOIL-1L TF-AA exhibited a slightly more pronounced effect than SHARPIN TF-AA (Fig. 2B). Introduction of both TF-AA mutants potentiated the suppression. NF-κB luciferase assays revealed that suppression of NF-κB was stronger in the presence of HOIL-1L TF-AA than in that of SHARPIN TF-AA and was further potentiated by the introduction of both TF-AA mutants (Fig. 2C). HOIL-1L TF-AA also suppressed expression of NF-κB target genes, and introduction of both TF-AA mutants further enhanced this suppression (Fig. 2D). These results indicated that HOIL-1L NZF is involved in NF-κB activation to a greater extent than SHARPIN NZF. Recruitment of LUBAC to the activated complex I precedes NF-κB activation [8]. We found that TNF-α-induced recruitment of LUBAC, as evaluated by the amount of HOIP, was suppressed in DKO MEFs with the TF-AA mutant of HOIL-1L or SHARPIN, and the recruitment was further suppressed in MEFs carrying both TF-AA mutants (Fig. 2E). The amounts of linear ubiquitin chains and HOIP in complex I rapidly decreased in a time-dependent manner in cells expressing HOIL-1L TF-AA, which implies that HOIL-1L NZF is involved in retention of LUBAC. In contrast to the decreased linear ubiquitination in complex I, RIPK1 ubiquitination was increased in MEFs co-expressing both TF-AA mutants as well as in those expressing the HOIL-1L TF-AA mutant.

To determine whether recognition of K63-linked or linear ubiquitin chains by SHARPIN NZF has different effects, we substituted the SHARPIN NZF domain with either the TAB2 NZF domain (SHARPIN TAB2 NZF) or the HOIL-1L NZF domain (SHARPIN HOIL-1L NZF), which preferentially bind to K63-linked or linear ubiquitin chains, respectively (Supplementary Fig. 1A) [23, 28]. The resulting chimeric SHARPIN proteins showed a preference for binding to either K63-linked or linear tetra-ubiquitin chains (Supplementary Fig. 1C). We then introduced the chimeric SHARPINs along with the TF-AA mutant of HOIL-1L into DKO MEFs (Fig. 2F). Although both SHARPIN TAB2 NZF and SHARPIN HOIL-1L NZF effectively enhanced NF-κB activation upon TNF-α stimulation compared with SHARPIN TF-AA, the effect was more pronounced for SHARPIN HOIL-1L NZF than for SHARPIN TAB2 NZF, despite subtle differences (Fig. 2G). Luciferase assays confirmed that SHARPIN HOIL-1L NZF contributed more effectively to NF-κB activation than SHARPIN TAB2 NZF (Fig. 2H), suggesting that binding to linear ubiquitin chains plays a major role in NF-κB activation. We next introduced the chimeric SHARPINs along with HOIL-1L WT into DKO MEFs (Supplementary Fig. 4A). Consistent with the results observed when introducing HOIL-1L TF-AA, SHARPIN HOIL-1L NZF restored NF-κB activation slightly more effectively than SHARPIN TAB2 NZF (Supplementary Fig. 4B). Luciferase assays demonstrated that the effect of SHARPIN HOIL-1L NZF on enhancing NF-κB activation was stronger than that of SHARPIN TAB2 NZF, although the difference was not statistically significant (Supplementary Fig. 4C). NF-κB activity was greater in cells expressing HOIL-1L WT than in those expressing HOIL-1L TF-AA, which confirmed the significant role of linear ubiquitin binding by HOIL-1L NZF in increasing NF-κB activation. Collectively, these findings suggest that the NZF domains of HOIL-1L and SHARPIN act cooperatively in the activation of the NF-κB pathway, and linear ubiquitin chains might play a dominant role over K63-linked ubiquitin binding.

### The NZF domain in HOIL-1L contributes to LUBAC-mediated cell death protection in concert with the NZF domain in SHARPIN

**Fig. 3:**
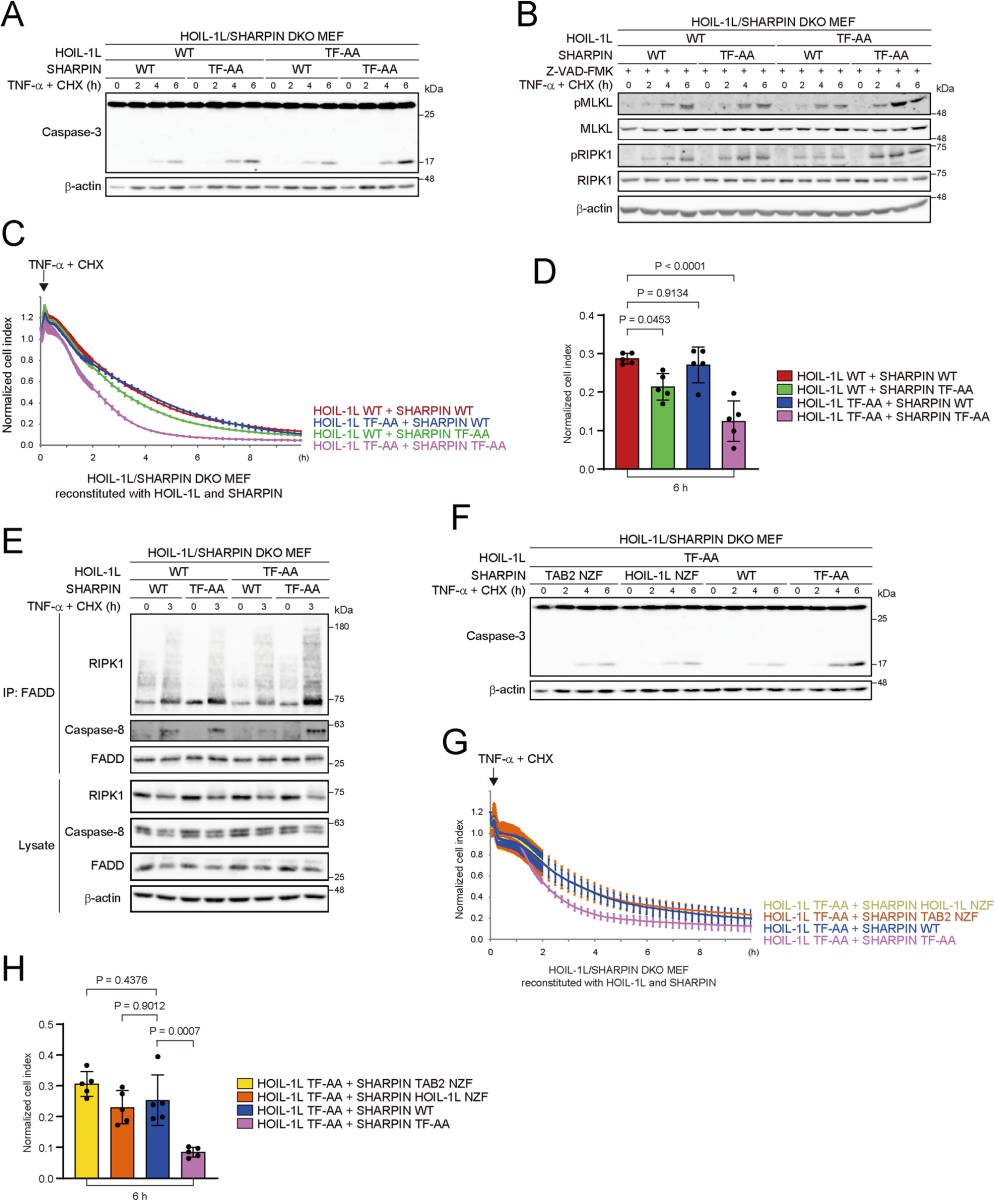
The NZF domain of HOIL-1L contributes to LUBAC-mediated cell death protection in concert with the NZF domain of SHARPIN. **A, F** HOIL-1L/SHARPIN DKO MEFs stably reconstituted with the indicated proteins were stimulated with TNF-α (2.5 ng/mL) and CHX (20 μg/mL) for the indicated times and analyzed by immunoblotting with the indicated antibodies. Data are representative of three independent experiments. **B** HOIL-1L/SHARPIN DKO MEFs stably reconstituted with the indicated proteins were stimulated with TNF-α (2.5 ng/mL) and CHX (20 μg/mL) for the indicated times after pretreatment with Z-VAD-FMK (10 μM) for 1 h, and analyzed by immunoblotting with the indicated antibodies. Data are representative of three independent experiments. **C, D, G, H** HOIL-1L/SHARPIN DKO MEFs stably reconstituted with the indicated proteins were stimulated with TNF-α (2.5 ng/mL) and CHX (20 μg/mL), and cell viability was continuously measured using the xCELLigence system. A representative image (**C, G**) and statistical analyses of the normalized cell index at 6 h (**D, H**). Data are shown as the mean ± s.d. of five independent experiments. *P* values were calculated by a one-way ANOVA with Tukey’s post hoc test. **E** HOIL-1L/SHARPIN DKO MEFs stably reconstituted with the indicated proteins were stimulated with TNF-α (2.5 ng/mL) and CHX (20 μg/mL) for the indicated times after pretreatment with Z-VAD-FMK (10 μM) for 1 h. Lysates were immunoprecipitated with anti-FADD antibody and immunoblotted as indicated. The experiments were repeated three times, independently, with similar results. Uncropped western blots are available for this figure in the Supplementary Material.

We next investigated the cooperative effect of the NZF domains on protection against cell death. Treatment of DKO MEFs expressing either the WT or TF-AA mutant of HOIL-1L and SHARPIN with TNF-α and CHX showed that loss of ubiquitin-binding activity in SHARPIN NZF increased cleavage of caspase-3 as previously reported [24], and loss of ubiquitin-binding activity in HOIL-1L NZF further potentiated cell death (both apoptosis and necroptosis) in MEFs expressing SHARPIN TF-AA (Fig. 3A–D, Supplementary Fig. 5A, B). Consistent results were obtained upon treatment with TNF-α and SM-164 (Supplementary Fig. 5C–E). The formation of complex II triggers TNF-α-induced cell death [21]. The generation of complex II was accelerated in DKO MEFs expressing SHARPIN TF-AA, and co-expression of HOIL-1L TF-AA enhanced this effect (Fig. 3E).

We showed that linear ubiquitin-binding by SHARPIN NZF plays a predominant role in NF-κB activation; therefore, we examined whether K63-linked or linear ubiquitin chains mediate the effect of SHARPIN NZF on cell death protection. When co-introduced into DKO MEFs together with HOIL-1L TF-AA, both SHARPIN TAB2 NZF and SHARPIN HOIL-1L NZF suppressed cell death almost equally (Fig. 3F–H). These results indicate that both K63-linked and linear chains are implicated in the cell death protection.

### Differential involvement of HOIL-1L NZF and SHARPIN NZF in NF-κB activation and cell death protection

We observed that linear ubiquitin-binding by HOIL-1L NZF is mainly involved in LUBAC-mediated NF-κB activation, and SHARPIN NZF contributes to this effect mainly through linear ubiquitin binding; in addition, both NZFs are cooperatively involved in protection against cell death, although SHARPIN NZF is more effective in this respect than HOIL-1L NZF. LUBAC is involved in tumorigenesis, and several compounds targeting LUBAC have demonstrated effectiveness [29–32]. However, attenuated LUBAC functions lead to inflammatory diseases [16, 22, 33–36]. By contrast, mice with mutations targeting HOIL-1L NZF develop normally under basal conditions [25].

**Fig. 4:**
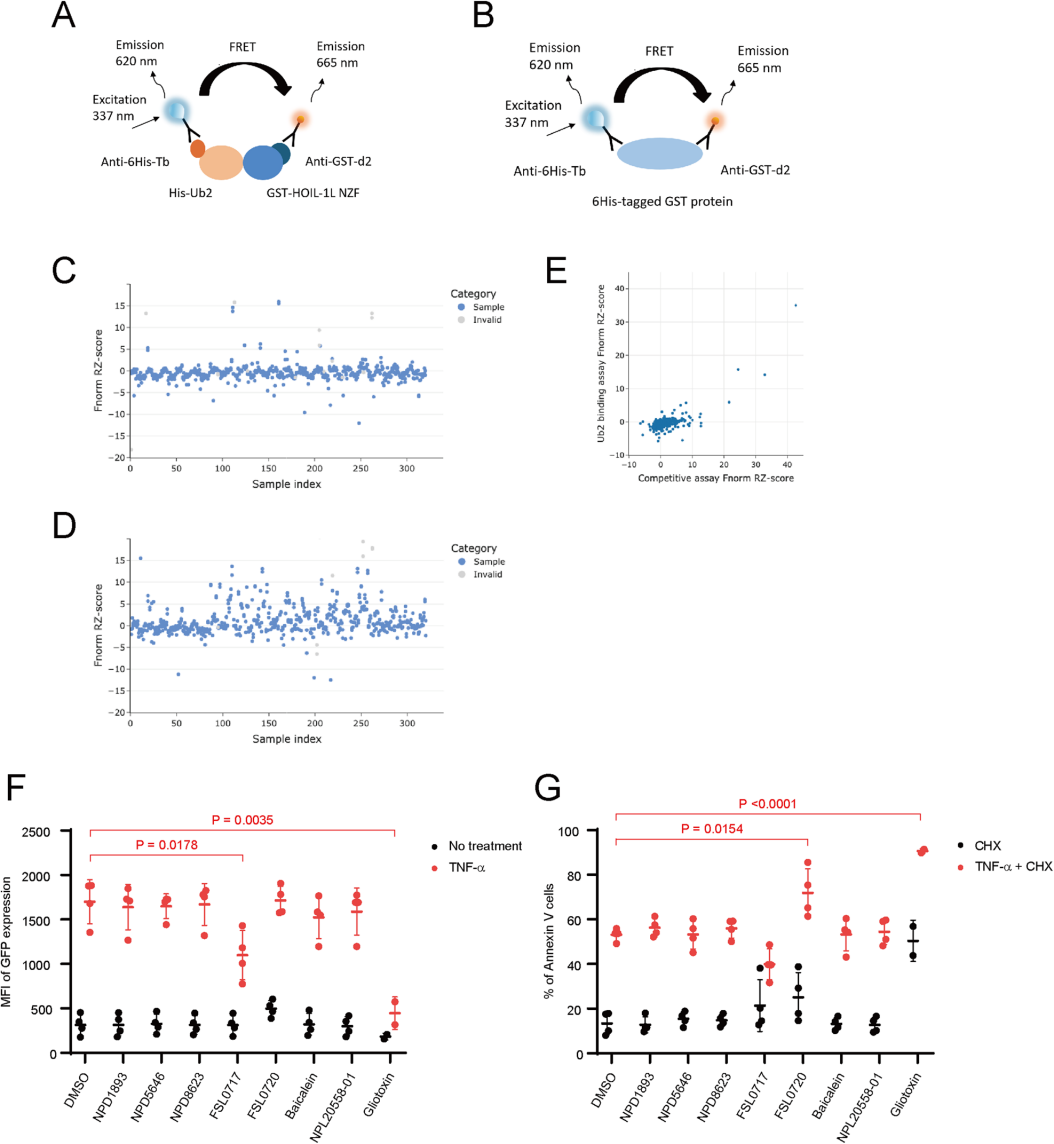
Screening of HOIL-1L NZF inhibitors. **A**, **B** Schematic illustration of the TR-FRET assay for detection of GST-tagged HOIL-1L NZF and His-tagged linear di-ubiquitin interactions (**A**). Counter TR-FRET assay with 6His-tagged GST protein (**B**). **C**, **D**, **E** Microscale thermophoresis (MST) assay of binding between HOIL-1L NZF and linear di-ubiquitin in the presence of compounds (**C**) and between compounds and linear di-ubiquitin (**D**). Correlation between **C** and **D** (**E**). **F**, **G** JR-GFP cells were treated with DMSO or the indicated compounds (10 μM) for 1 h and then stimulated with TNF-α (5 ng/mL) for 4 h (**F**) or TNF-α (5 ng/mL) plus CHX (20 μg/mL) for 6 h (**G**). Samples were analyzed by flow cytometry after Annexin V staining. Data are displayed as the mean fluorescence intensity (MFI) of GFP expression (**F**) and the frequency of Annexin V-positive cells (**G**). Data are shown as the mean ± s.d. of four independent experiments. *P* values are from a two-tailed Student’s *t* test.

**Fig. 5:**
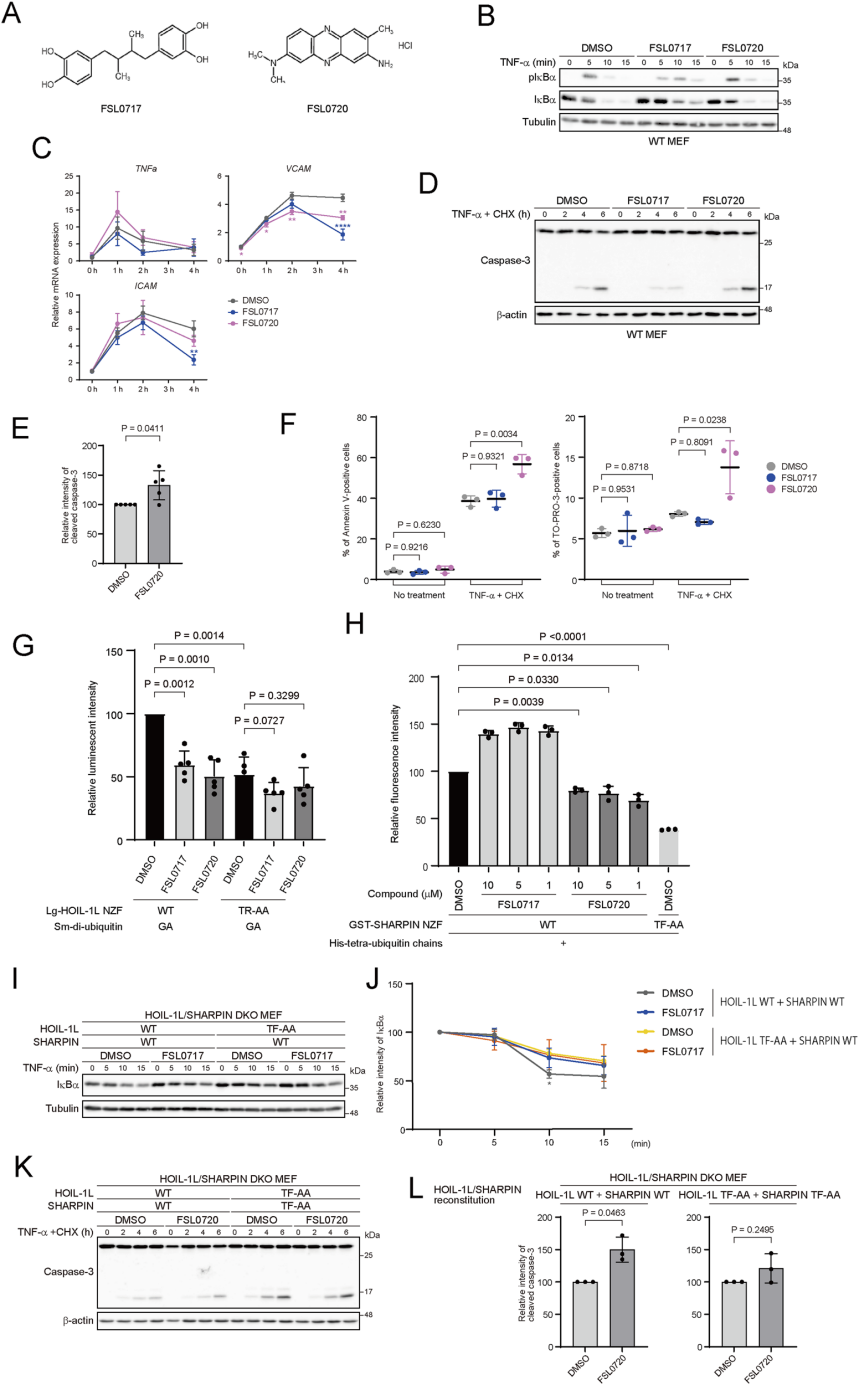
FSL0717 suppressed NF-κB activation by inhibiting the HOIL-1L NZF, whereas FSL0720 increased cell death by inhibiting both the HOIL-1L NZF and SHARPIN NZF. **A** Chemical structures of the identified compounds. **B**, **D**, **E** WT MEFs were stimulated with TNF-α (1 ng/mL) (**B**) or TNF-α (2.5 ng/mL) plus CHX (20 μg/mL) (**D**) for the indicated times after treatment with DMSO or the indicated compounds (10 μM) for 1 h and analyzed by immunoblotting with the indicated antibodies. Quantitative analysis of cleaved caspase-3 shown in **D** was performed at 6 h after stimulation (**E**). Data are representative of three (**B**) or five (**D**) independent experiments and are shown as the mean ± s.d. (**E**). *P* values were obtained using a one-sample *t* test. **C** HOIL-1L/SHARPIN DKO MEFs stably reconstituted with the indicated proteins were stimulated with TNF-α (1 ng/mL) for the indicated time after treatment with DMSO or the indicated compounds (10 μM), and expression of NF-κB target genes was measured by quantitative PCR. All gene expression levels are normalized against the corresponding levels of *ACTB* and expressed as fold changes relative to DMSO-treated cells at 0 h. Data are shown as the mean ± s.d. of three independent experiments. *P* values were calculated by a one-way ANOVA with Tukey’s post hoc test. *P < 0.05, **P < 0.01, and ****P < 0.0001. **F** JR-GFP cells were treated with DMSO or the indicated compounds (10 μM) for 1 h and stimulated with TNF-α (5 ng/mL) plus CHX (20 μg/mL) for 0 or 6 h. Samples were analyzed by flow cytometry after Annexin V and TO-PRO-3 staining. Data are displayed as the frequencies of Annexin V-positive cells (left) and TO-PRO-3-positive cells (right). Data are shown as the mean ± s.d. of three independent experiments. *P* values were calculated by a one-way ANOVA with Tukey’s post hoc test. **G** HEK293T cells were transiently transfected with Sm-di-ubiquitin (GA mutant) and Lg-HOIL-1L NZF (WT or TR-AA mutant), cultured for 24 h, and then treated with DMSO or the indicated compounds (10 μM) for 1 h. Luminescence intensity was measured using a NanoBiT system. Data are shown as the mean ± s.d. of five independent experiments. *P* values were obtained using a one-sample *t* test (Lg-HOIL-1L NZF WT) or a two-tailed Student’s *t* test (Lg-HOIL-1L TR-AA mutant). **H** His-tagged di-ubiquitin, GST-tagged SHARPIN NZF, and the indicated compounds were mixed and incubated for 1 h. Fluorescence intensity was measured using a FRET assay. Data are shown as the mean ± s.d. of three independent experiments. *P* values are from a one-sample *t* test. **I**, **J** HOIL-1L/SHARPIN DKO MEFs stably reconstituted with the indicated proteins were stimulated with TNF-α (1 ng/mL) for the indicated times after treatment with DMSO or the indicated compounds (10 μM) for 1 h, and analyzed by immunoblotting with the indicated antibodies (**I**). Quantitative analysis of IκBα was performed, with all data normalized against the corresponding values at 0 min (**J**). Data are representative of four independent experiments (**I**) and shown as the mean ± s.d. (**J**). *P* values were obtained using a one-way ANOVA with Tukey’s post hoc test. *P < 0.05. **K, L** HOIL-1L/SHARPIN DKO MEFs stably reconstituted with the indicated proteins were stimulated with TNF-α (2.5 ng/mL) plus CHX (20 μg/mL) for the indicated times after treatment with DMSO or the indicated compounds (10 μM) for 1 h and analyzed by immunoblotting with the indicated antibodies. Quantitative analysis of cleaved caspase-3 was performed at 6 h after stimulation (**L**). Data are representative of three independent experiments (**K**) and shown as the mean ± s.d. (**L**). *P* values were obtained using a one-sample *t* test. Uncropped western blots are available for this figure in the Supplementary Material.

We then tried to identify small compounds capable of inhibiting the interaction between HOIL-1L NZF and linear ubiquitin chains. To achieve this, we established a high-throughput time-resolved fluorescence resonance energy transfer (TR-FRET) assay to screen for compounds capable of inhibiting binding between glutathione S-transferase (GST)-tagged HOIL-1L NZF and His-tagged linear di-ubiquitin (Fig. 4A). We screened 33,858 small molecules, including U.S. Food and Drug Administration-approved drugs, natural and synthetic compounds, and compounds of the DDI Core library. “Hit” compounds from the screen that inhibited the TR-FRET of His-tagged GST were excluded (Fig. 4B, Supplementary Table). A total of 320 “Hit” compounds in the high-throughput screening were further evaluated for their ability to inhibit binding between HOIL-1L NZF and linear di-ubiquitin using a microscale thermophoresis assay, whereas compounds binding to linear di-ubiquitin were eliminated (Fig. 4C–E). Then, seven selected compounds were evaluated using JR-GFP cells, in which GFP expression is induced by NF-κB activation [37] (Supplementary Fig. 6A, B). The assay was validated using gliotoxin, a known LUBAC inhibitor [38] (Fig. 4F, G). FSL0717, also known as nordihydroguaiaretic acid, attenuated TNF-α-induced NF-κB activation without increasing apoptosis, whereas FSL0720, also known as neutral red, increased apoptosis induced by TNF-α plus CHX without overtly affecting NF-κB activation (Figs. 4F, G, 5A). These findings were confirmed in WT MEFs as well as in JR-GFP cells (Fig. 5B–F). To further dissect the mechanism underlying differential effects of FSL0717 and FSL0720, we evaluated whether both compounds indeed inhibited the interaction between HOIL-1L NZF and linear ubiquitin chains in living cells. We established a NanoLuc Binary Technology (NanoBiT) assay in HEK293T cells. Because ubiquitin chains are easily disassembled by deubiquitinating enzymes in cells [39, 40], we utilized the linear di-ubiquitin GA mutant, in which Gly76 of distal ubiquitin is mutated to Ala (linear di-Ub GA), to avoid cleavage by these enzymes (Supplementary Fig. 7A–D). Evaluation of the interaction between HOIL-1L NZF and linear di-Ub GA showed that both compounds inhibited binding between HOIL-1L NZF WT and linear di-ubiquitin in living cells (Fig. 5G). We speculated that the increased cell death induced by FSL0720 may be related to its interaction with SHARPIN NZF. This was supported by a TR-FRET assay between SHARPIN NZF and linear tetra-ubiquitin chains, which showed that FSL0720, but not FSL0717, reduced the signal (Fig. 5H, Supplementary Fig. 8). The effects of both compounds were mitigated in the corresponding mutant cells, supporting their on-target effects (Fig. 5I–L). We further performed blind docking simulations using QuickVina-W [41] to evaluate binding of the compounds to the NZF domains. Both compounds are predicted to be located near F202 of HOIL-1L NZF, a critical residue for the interaction with ubiquitin chains (Supplementary Fig. 9, left). This suggests that binding of both compounds impairs the recognition of ubiquitin chains by perturbing the conformation of F202 and neighboring T201. In the docking simulation of SHARPIN NZF and FSL0720, bound FSL0720 was accessible to T351, which is critical for recognition of ubiquitin chains, suggesting that it may elicit inhibitory activity by perturbing the conformation of the critical residue as in the case of HOIL-1L NZF (Supplementary Fig. 9, right). However, the docking model for SHARPIN NZF should be interpreted with caution due to uncertainties with the predicted protein structure [42].

Taken together, these findings indicate that both HOIL-1L and SHARPIN NZF cooperatively play crucial roles in NF-κB activation and protection from regulated cell death. However, HOIL-1L NZF is mainly involved in NF-κB activation, whereas SHARPIN NZF is mainly involved in cell death protection.

## Discussion

The two accessory subunits of LUBAC, HOIL-1L and SHARPIN, play crucial roles in mediating its functions as well as in stabilizing the complex [11, 23, 24]. Here, we examined the effects of the NZF domains of both subunits on LUBAC functions and demonstrated that the simultaneous loss of ubiquitin-binding activity in both NZFs synergistically decreases the protection from cell death as well as NF-κB activation.

Loss of ubiquitin-binding activity in either HOIL-1L NZF or SHARPIN NZF reduced the interaction of LUBAC with complex I upon TNF-α stimulation, and loss of ubiquitin-binding activity in both domains further attenuated the interaction (Fig. 2E). Studies have suggested that the recruitment and presence of LUBAC in complex I are important for NF-κB activation and cell death protection [8, 18]: LUBAC is recruited to complex I via recognition of K63-linked ubiquitin chains [8, 18], and the linear ubiquitination activity of LUBAC is necessary for the sustained stability of complex I [43]. Recognition of K63-linked ubiquitin chains by SHARPIN NZF mediates the recruitment of LUBAC [24], whereas HOIL-1L NZF may not be involved in the recruitment given that linear ubiquitin chains cannot be conjugated to complex I in the absence of LUBAC recruitment. However, upon recruitment, LUBAC conjugates linear ubiquitin chains to the components of complex I [8, 20, 44]. It is thus likely that the NZF domains of both HOIL-1L and SHARPIN are involved in the sustained recruitment of LUBAC to complex I by recognizing linear ubiquitin chains. Consequently, loss of the ubiquitin-binding activity of both NZF domains synergistically attenuates NF-κB activation as well as cell death protection.

Although both NZF domains cooperatively regulate the functions of LUBAC, each NZF domain exhibits distinct roles in NF-κB activation and cell death protection, as demonstrated in cells expressing the NZF mutant of either subunit. The results showed that HOIL-1L NZF is more profoundly involved in NF-κB activation than SHARPIN NZF (Fig. 2B–D). Since SHARPIN NZF binds to both K63-linked and linear ubiquitin chains, we generated SHARPIN mutants in which SHARPIN NZF was replaced by HOIL-1L NZF or TAB2 NZF, which preferentially recognize linear or K63-linked ubiquitin chains, respectively, to examine the roles of binding to each type of ubiquitin chain. We found that the SHARPIN HOIL-1L NZF increased NF-κB activation more effectively than SHARPIN WT. Considering that HOIL-1L NZF binds to linear ubiquitin chains more robustly than SHARPIN NZF [23], it seems plausible that the binding to linear chains within LUBAC plays crucial roles in NF-κB activation. Binding to linear ubiquitin chains helps retain LUBAC at complex I, facilitating the attachment of linear ubiquitin chains to NEMO and thereby promoting NF-κB activation. Our observation implied that binding of LUBAC to linear chains facilitates linear ubiquitination by HOIP in complex I by facilitating retention of LUBAC at the complex; therefore, HOIL-1L NZF plays major roles in NF-κB activation.

In contrast to NF-κB activation, SHARPIN NZF plays a prominent role in cell death protection (Fig. 3A–D). Replacement of SHARPIN NZF with other NZFs that preferentially bind to K63-linked or linear ubiquitin chains revealed that both K63-linked and linear ubiquitin chains are relevant to cell death protection. Additionally, the ubiquitin-binding activity of HOIL-1L has a protective function against cell death in concert with that of SHARPIN (Fig. 3A–E). Loss of ubiquitin-binding activity of HOIL-1L rendered cells mildly sensitive to death induced by TNF-α and SM-164, which inhibits generation of K63-linked chains by cIAP1/2; therefore, it seems likely that HOIL-1L protects against cell death mainly by retaining LUBAC at complex I via recognizing linear ubiquitin chains after it is recruited to the complex. Therefore, SHARPIN may play a predominant role in cell death protection by inducing both recruitment and retention of LUBAC to complex I, while HOIL-1L is involved in cell death protection by inducing prolonged retention. A HOIL-1L NZF mutant lacking the ubiquitin-binding activity exacerbates the skin inflammation phenotype of SHARPIN-deficient mice (cpdm mice) [25]. Lack of SHARPIN markedly decreases the amount of LUBAC [12, 13, 25]; however, introduction of the HOIL-1L NZF mutant does not affect the amount of LUBAC [25] (Fig. 1B). Because increased keratinocyte apoptosis underlies the exacerbation of dermatitis [14, 33–36], the observation confirms the involvement of ubiquitin binding by HOIL-1L NZF in cell death protection. We also observed that RIPK1 ubiquitination was increased in MEFs co-expressing both TF-AA mutants as well as in those expressing the HOIL-1L TF-AA mutant, despite the decrease in linear ubiquitination in complex I. Given the critical role of RIPK1 ubiquitination in regulating cell death [17, 45], this increase may influence cell death protection.

We identified two compounds, FSL0717 and FSL0720, that inhibited binding between HOIL-1L NZF and linear ubiquitin chains in living cells; however, their effects differed in relation to NF-κB activation and cell death (Figs. 4, 5). The distinct roles of the NZF domains in HOIL-1L and SHARPIN (Figs. 2, 3) suggest that these effects are related to the interaction with SHARPIN NZF. This was supported by the inhibitory effect of FSL0720 on binding between SHARPIN NZF and linear ubiquitin chains (Fig. 5H). We suspect that FSL0720 also inhibits the interaction between SHARPIN NZF and K63-linked ubiquitin chains, although this was not extensively tested. FSL0720 (neutral red) is commonly used for cell viability analyses, whereas FSL0717 (nordihydroguaiaretic acid) affects various molecules such as lipoxygenase, KEAP1, and NRF2 [46, 47], although their effects on LUBAC have not been reported.

In conclusion, comprehensive analysis of the NZF domains of HOIL-1L and SHARPIN indicated that the two NZF domains are cooperatively involved in both NF-κB activation and cell death protection. Binding to linear ubiquitin chains functions in NF-κB activation probably by maintaining the recruitment of LUBAC to complex I, whereas ubiquitin binding by SHARPIN plays predominant roles in cell death protection, although HOIL-1L NZF is also involved in this function. The identification of a small molecule that inhibits the ubiquitin-binding activity of both HOIL-1L NZF and SHARPIN NZF provides a potential therapeutic strategy for targeting elevated LUBAC activity in diseases such as cancer.

## Supplementary information

Supplementary Materials

Reproducibility-checklist

## Data Availability

All data are available in the article and the supplementary information, and from the corresponding author upon reasonable request.
