# Supplementary material for: Synergistic involvement of the NZF domains of the LUBAC accessory subunits HOIL-1L and SHARPIN in the regulation of LUBAC function
Source: Cell Death Dis. 2024 Nov 11;15(11):813. doi: 10.1038/s41419-024-07199-z (PMC11555115; doi:10.1038/s41419-024-07199-z)
Supplement: Supplementary file 1 — Supplementary Materials [file 41419_2024_7199_MOESM1_ESM.docx]

SUPPLEMENTARY MATERIALS

**Synergistic involvement of the NZF domains of the LUBAC accessory subunits HOIL-1L and SHARPIN in the regulation of LUBAC function**

Yusuke Toda, Hiroaki Fujita, Koshiki Mino, Takuto Koyama, Seiji Matsuoka, Toshie Kaizuka, Mari Agawa, Shigeyuki Matsumoto, Akiko Idei, Momoko Nishikori, Yasushi Okuno, Hiroyuki Osada, Minoru Yoshida, Akifumi Takaori-Kondo, and Kazuhiro Iwai

**Materials and methods**

**Plasmids**

Complementary DNAs (cDNAs) used in this study were described previously [1-3]. Mutants of mouse HOIL-1L (T201A/F202A), mouse SHARPIN (T351A/F352A), human HOIL-1L (T203A/R210A), human SHARPIN (T358A/F359A), and linear di-ubiquitin (G76V, G76A, G75A/G76A, G75V/G76V, L73P) were generated by PCR. Mutants of mouse SHARPIN, in which the NZF domain was replaced with the NZF domain of TAB2 or HOIL-1L, were generated from the amplified ORFs of mouse TAB2 and mouse HOIL-1L. The cDNAs were ligated to the appropriate epitope-tagged sequences and cloned into the pcDNA3.1, pMXs-IRES-puro-att, pMXs-IRES-Bsr-att [4], pBiT1.1, pBiT2.1 (Promega), pT7-7, pGEX6p-1, and pMAL-c2X vectors. An sgRNA targeting HOIL-1L (5′-CGTGCTCGCTCTTCCTCGTC-3′) was cloned into pSpCas9(BB)-2A-GFP (PX458) (Addgene) to generate HOIL-1L knockout cells.

**Antibodies and reagents**

The following antibodies were used for immunoblotting: anti-IκBα (Cell Signaling Technology 9242; Cat, 1:2000 dilution), anti-pIκBα (Cell Signaling Technology 9246; 1:2000 dilution), anti-caspase-3 (Cell Signaling Technology 9662, 1:2000 dilution), anti-caspase-8 (Cell Signaling Technology 4790, 1:2000 dilution), anti-RIP (Cell Signaling Technology 3493, 1:2000 dilution), anti-pRIP (Cell Signaling Technology 31122, 1:2000 dilution), anti-TNFR1 (Cell Signaling Technology 13377, 1:2000 dilution), HRP-linked anti-mouse IgG (Cell Signaling Technology 7076, 1:10000 dilution), anti-β-actin (Sigma-Aldrich A5316, 1:2000 dilution), anti-MLKL (Sigma-Aldrich SAB1302339, 1:2000 dilution), anti-linear ubiquitin (Sigma-Aldrich MABS199, 1:2000 dilution), anti-DDDDK (MBL PM020, 1:2000), anti-His (MBL D291-3, 1:2000 dilution), HRP-linked anti-rabbit (Cytiva NA934V, 1:10000), anti-ubiquitin (Santa Cruz Biotechnology sc-8017, 1:2000 dilution), anti-tubulin (CEDARLANE CLT9002, 1:2000 dilution), anti-pMLKL (Abcam ab196436, 1:2000 dilution), anti-FADD (Enzo Life Sciences ADI-AAM-212-E, 1:2000 dilution), anti-HOIP (our laboratory, 1:2000 dilution), anti-HOIL-1L (our laboratory, 1:2000 dilution), and anti-SHARPIN (our laboratory, 1:2000 dilution). Anti-FLAG (Sigma-Aldrich F3165) and anti-FADD (Santa Cruz Biotechnology sc-6036) were used for immunoprecipitation. Anti-Annexin V (Biolegend 640924, 1:200 dilution) was used for flow cytometry analysis.

The following reagents were used in this study: recombinant mouse TNF-α (R&D Systems 410-MT), recombinant human TNF-α (Fujifilm Wako Pure Chemical Corporation 203-15263), cycloheximide (Calbiochem 239764), and SM-164 (Selleck S7089).

**Cell lines**

MEFs derived from WT, cpdm, and HOIL-1L-null mice were generated in our laboratory [2, 5]. HEK293T was a gift from Dr. Eijiro Nakamura. Plat-E was a gift from Dr. Toshio Kitamura. JR-GFP was a gift from Dr. Xin Lin. All cell lines except JR-GFP were cultured in Dulbecco’s Modified Eagle Medium (DMEM; Sigma-Aldrich) supplemented with 10% fetal bovine serum (FBS; Sigma-Aldrich), 100 IU/mL penicillin, and 100 mg/mL streptomycin at 37°C under 5% CO_2_. JR-GFP was cultured in RPMI 1640 medium (Sigma-Aldrich) supplemented with 10% FBS, 100 IU/mL penicillin, and 100 mg/mL streptomycin at 37°C under 5% CO_2_.

**Generation of CRISPR/Cas9-based KO cells**

To generate HOIL-1L and SHARPIN double knockout MEFs, a PX458 plasmid encoding sgRNA targeting HOIL-1L was transfected into MEFs from cpdm mice by electroporation. After 24 h, GFP-expressing cells were isolated using a FACS Aria III sorter (BD Biosciences). Subsequently, they were plated at low density, and single colonies were picked using cloning rings.

**Retroviral infections and generation of stable cell lines**

Appropriate pMXs plasmids were transfected into Plat-E packaging cells using Lipofectamine 2000 (Thermo Fisher Scientific). After 48 h, the retroviral supernatant was collected and passed through a 0.45 mm filter. MEFs were infected with retrovirus in the presence of 10 μg/mL polybrene (Merk Millipore) for 16 h. The stably infected cells were selected using puromycin (Sigma-Aldrich) or blasticidin (InvivoGen).

**Protein expression and purification**

Proteins were expressed and purified from *Escherichia coli* BL21-CodonPlus (DE3)-RIPL. To generate GST-hHOIL-1L NZF (WT, TR-AA) and GST-hSHARPIN NZF (WT, TF-AA), bacteria were grown at 30°C until OD600 reached 0.6. Protein expression was induced by addition of IPTG, and the culture was continued at 15°C overnight. Bacteria were collected by centrifugation. The bacterial pellet was suspended in GST lysis buffer (20 mM Tris–HCl pH 7.5 and 200 mM NaCl) supplemented with 2 mM phenylmethylsulfonyl fluoride (PMSF), 1 mM DTT, and a protease inhibitor cocktail (Roche), and lysed by sonication. The lysate was clarified by centrifugation at 20,000 g for 20 min at 4°C, and incubated with Glutathione Sepharose 4 Fast Flow (Cytiva) for 1.5 h at 4°C. The beads were washed five times with GST lysis buffer, and GST protein was eluted with glutathione buffer (20 mM glutathione, 20 mM Tris–HCl pH 7.5, and 200 mM NaCl). The sample was loaded onto a PD-10 column (GE Healthcare) and eluted using replacement buffer (20 mM Tris-HCl pH 7.5, 1 mM DTT).

To generate MBP-His-mHOIL-1L (WT and TF-AA) and MBP-His-mSHARPIN (WT, TF-AA, TAB2 NZF, and HOIL-1L NZF), bacteria were grown at 30°C until OD600 reached 0.6. Protein expression was induced by addition of IPTG, and the culture was continued at 15°C overnight. Bacteria were collected by centrifugation. The bacterial pellet was suspended in GST lysis buffer supplemented with 2 mM PMSF, 1 mM DTT, and a protease inhibitor cocktail (Roche) and lysed by sonication. The lysate was clarified by centrifugation at 20,000 g for 20 min at 4°C and incubated with amylose resin (New England BioLabs) for 4 h at 4°C. The beads were washed with GST lysis buffer five times, and the MBP protein was eluted with maltose buffer (10 mM maltose, 20 mM Tris–HCl pH 7.5, and 200 mM NaCl).

To generate His-linear di-ubiquitin (WT, GV, AA, GA, L73P, and VV), bacteria were grown at 30°C until OD600 reached 0.6. Protein expression was induced by addition of IPTG, and the culture was continued at 15°C overnight. Bacteria were collected by centrifugation. The bacterial pellet was suspended in His lysis buffer (50 mM Tris–HCl pH 8.0 and 150 mM NaCl) supplemented with 2 mM PMSF, 5 mM 2-mercaptoethanol, and a protease inhibitor cocktail (Roche) and lysed by sonication. The lysate was clarified by centrifugation at 20,000 g for 20 min at 4°C and incubated with Ni-NTA Agarose (QIAGEN) for 1.5 h at 4°C. The beads were washed three times with His lysis buffer supplemented with 10 mM imidazole and twice with His lysis buffer supplemented with 20 mM imidazole, and the His protein was eluted with imidazole buffer (300 mM imidazole, 50 mM Tris–HCl pH 8.0, and 150 mM NaCl). The sample was dialyzed against replacement buffer using Slide-A-Lyzer MINI Dialysis Devices (Thermo Fisher Scientific).

**Immunoblotting**

Cells were lysed in lysis buffer containing 50 mM Tris-HCl pH 7.5, 150 mM NaCl, 1% Triton X-100, 2 mM PMSF, a protease inhibitor cocktail (Sigma-Aldrich), and a phosphatase inhibitor cocktail (Nacalai Tesque). After incubation on ice for 20 min, the lysates were clarified by centrifugation at 20,000 g for 20 min at 4°C. The lysates were denatured in 1× sample buffer (2% SDS, 10% glycerol, 0.1% bromophenol blue, 50 mM Tris-HCl pH 6.8, and 100 mM DTT), and then boiled at 95°C for 5 min. Samples were resolved by sodium dodecyl sulfate-polyacrylamide gel electrophoresis (SDS-PAGE) and transferred to polyvinylidene difluoride membranes. After blocking in Tris-buffered saline containing 0.1% Tween-20 and 5% (w/v) nonfat dry milk, the membranes were incubated with the appropriate primary antibodies, followed by the corresponding secondary antibodies. The bound antibodies were visualized by enhanced chemiluminescence and analyzed on a LAS4000mini instrument (GE Healthcare).

**Quantitative PCR**

RNA was isolated using an RNeasy Mini kit (QIAGEN). DNase-treated RNA was reverse-transcribed into cDNA using a High-Capacity RNA-to-cDNA Kit (Thermo Fisher Scientific). Real-time PCR was performed using Power SYBR Green PCR Master Mix (Applied Biosystems) on a ViiA7 Real-Time PCR System (Applied Biosystems). All gene expression levels were normalized against the corresponding levels of *ACTB*. The primers used for quantitative PCR were as follows: *TNFa*_Forward, 5′-GGTGCCTATGTCTCAGCCTCTT-3′; *TNFa*_Reverse, 5′-GCCATAGAACTGATGAGAGGGAG-3′; *VCAM*_Forward, 5′-GCTATGAGGATGGAAGACTCTGG-3′; *VCAM*_Reverse, 5′-ACTTGTGCAGCCACCTGAGATC-3′; *ICAM*_Forward, 5′-AAACCAGACCCTGGAACTGCAC-3′; *ICAM*_Reverse, 5′-GCCTGGCATTTCAGAGTCTGCT-3′; *IL-6*_Forward, 5′-TACCACTTCACAAGTCGGAGGC-3′; *IL-6*_Reverse, 5′-CTGCAAGTGCATCATCGTTGTTC-3′; *ACTB*_Forward, 5′-CATTGCTGACAGGATGCAGAAGG-3′; and *ACTB*_Reverse, 5′-TGCTGGAAGGTGGACAGTGAGG-3′.

**Cell viability assay**

Cell viability was measured as an impedance-based cell index using the xCELLigence system (Agilent Technologies). For each sample, 2 × 10^4^ cells were seeded in an E-Plate 16 PET (Agilent Technologies). The next day, cells were treated with TNF-α (2.5 ng/mL) plus CHX (20 μg/mL) or TNF-α (2.5 ng/mL) plus SM-164 (200 nM), and the cell index was continuously monitored. Data were normalized against cell indices at the time of TNF-α plus CHX or TNF-α plus SM-164 treatment.

**Luciferase assay**

HEK293T cells were transfected with pGL4.32 (Luc2p/NF-kB-RE/Hygro) and pGL4.74 (hRLuc/TK) (Promega) along with plasmids encoding WT or mutant LUBAC components using Lipofectamine 2000. After culturing for 24 h, cells were lysed, and luciferase activity was measured using a Dual-Luciferase Reporter Assay system (Promega) on a Lumat Luminometer (Berthold).

**Immunoprecipitation of TNFR1 signaling complex (complex I)**

After stimulation with FLAG-His-TNF-α (1 μg/mL), cells were lysed in lysis buffer containing 10 mM Tris-HCl (pH 7.5), 150 mM NaCl, 0.2% NP-40, 10% glycerol, 2 mM PMSF, a protease inhibitor cocktail (Sigma-Aldrich), a phosphatase inhibitor cocktail (Nacalai Tesque), and 5 mM N-ethylmaleimide. Following addition of 7.5 mM cysteine, lysates were clarified by centrifugation at 10,000 g for 20 min at 4°C. TNFR1 signaling complex 1 was immunoprecipitated with anti-FLAG M2 antibodies conjugated with Dynabeads protein G (Invitrogen) for 75 min at 4°C with rotation. The precipitates were washed five times with lysis buffer. TNFR complex 1 was eluted with 400 ng/mL of 3× FLAG peptide (Sigma-Aldrich) in 30 μL TBS and analyzed by immunoblotting.

**Immunoprecipitation of a cytosolic complex (complex II)**

After pretreatment with Z-VAD-FMK (PEPTIDE) for 60 min, cells were stimulated with TNF-α (2.5 ng/mL) and CHX (20 μg/mL) for 2 or 3 h. Cells were lysed in lysis buffer containing 30 mM Tris-HCl pH 7.5, 120 mM NaCl, 1% Triton X-100, 10% glycerol, 2 mM PMSF, and a protease inhibitor cocktail (Sigma-Aldrich), followed by centrifugation at 10,000 g for 20 min at 4°C. The clarified lysates were incubated with anti-FADD antibodies conjugated with Dynabeads protein A (Invitrogen) at 4°C overnight with rotation. The precipitates were washed three times with lysis buffer and once with PBS. Proteins were eluted using 1× sample buffer, boiled at 95°C for 5 min, and analyzed by immunoblotting.

**High throughput screening**

A high throughput screen for inhibitors of HOIL-1L was performed using the TR-FRET assay. For the primary screen, 20,393 compounds of the RIKEN Natural Product Depository (NPDepo) library provided by the Drug Discovery Chemical Bank Unit, RIKEN; 9,600 compounds from the Drug Discovery Initiative, the University of Tokyo; and 3,865 compounds from FDA-approved drug libraries were incubated in 20 μL of the TR-FRET assay mixture (DMSO at a final concentration of 0.5%). The assay buffer consisted of 20 mM Tris-HCl pH 7.5, 0.1% BSA, and 0.01% Tween 20. A volume of 0.1 μL of compound solution was added into each well of an OptiPlate-384 (6007299, PerkinElmer), and 5 μL of assay buffer containing 20 nM His-tagged linear di-ubiquitin was added. The plates were incubated at room temperature for approximately 30 min. Then, 5 μL of assay buffer containing 20 nM GST-tagged HOIL-1L NZF was added into each well, and the plates were incubated at 23°C for 1 h. A mixture of TR-FRET reagents was prepared by diluting each stock solution of MAb Anti-6HIS-Tb cryptate Gold (61HI2TLB, Cisbio Bioassays) and MAb Anti GST-d2 (61GSTDLB, Cisbio Bioassays) with assay buffer at final concentrations of 0.105 μg/mL and 2 μg/mL, respectively. Then, 10 μL of the TR-FRET reagent mixture was added into the wells using a Multidrop Combi reagent dispenser, and the plates were incubated at 23°C for 1 h. Fluorescence signals were detected in TR-FRET mode at wavelengths of 665 and 620 nm using an EnVision plate reader (PerkinElmer). A counter screen assay was performed in which the tagged HOIL-1L NZF and linear di-ubiquitin interaction pair was replaced by recombinant 6His-tagged GST protein (ab89494, Abcam) at a final concentration of 0.2 nM in the assay mixture (20 μl) described above. Data analyses were performed using a Genedata Screener software (Genedata). The percent inhibition for each sample was calculated as 100 × [1 − (sample-background)/(control-background)]. The 50% inhibitory concentration (IC50) for each compound was calculated by fitting dose-response data using the Smart Fit method in the software.

**Microscale thermophoresis (MST) binding assay**

Dye-labeled di-ubiquitin was prepared by mixing 200 μM di-ubiquitin with 200 μM RED-NHS dye (MO-L011, NanoTemper Technologies) in phosphate-buffered saline and incubating in the dark at 23°C for 30 min. Excess RED-NHS dye was removed by centrifugal filters (UFC501096, Merck). MST binding assays were performed using Dianthus NT.23 PicoDuo (NanoTemper Technologies) with a laser irradiation time of 5 sec and pico mode off. Di-ubiquitin binding assays were performed using sample solutions containing 10 nM dye-labeled di-ubiquitin, 10 mM HEPES-NaOH, 5 mM glutathione, 0.005% Tween20, and 2% DMSO adjusted to pH 7.4. For the competitive assay, 0.5 μM GST-tagged human HOIL-1L was added to the solution described above. Data analyses were performed with DI.Control Software (NanoTemper Technologies) and Python 3.10.6 environment. The obtained Fnorm values (normalized fluorescence intensity ratio before and after laser emission) were normalized to the RZ’-score [ (sample Fnorm – DMSO control Fnorm median) * 0.67 / DMSO control median absolute deviation] and used as hit criteria. Samples with initial fluorescence values that were not in the range of 80–150 or with annotation flags that indicated aggregation or well-scan anomaly indicated by DI.Control software were excluded from the analysis. Dissociation constants of the active compounds were determined using a curve fitting model with the Hill coefficient fixed to 1 using Genedata Screener (Genedata).

**Flow cytometry analysis**

Cells were harvested and stained with fluorochrome-conjugated antibodies after washing. Flow cytometry data were acquired on a FACSCanto II (BD Biosciences) with FACS Diva software v. 6.1.2 (BD Biosciences), and the results were analyzed using FlowJo software v. 9.9.6 (BD Biosciences).

**NanoBiT assay**

Human HOIL-1L NZF (WT or TR-AA) fused to N-terminal LgBiT and linear di-ubiquitin fused to C-terminal SmBiT were selected as the optimal combinations after screening eight possible combinations (each protein was fused either at the N or C terminus with LgBiT and SmBiT). HEK293T cells were plated at a density of 1 × 10^4^ in a white ViewPlate-96 TC (PerkinElmer) with FluoroBrite DMEM (Thermo Fisher Scientific) supplemented with 10% FBS and 4 mM l-glutamine (Fujifilm Wako Pure Chemical Corporation). The next day, the cells were transfected with pBiT1.1 and pBiT2.1 plasmids encoding LgBiT-fused HOIL-1L NZF and SmBiT-fused linear di-ubiquitin, respectively, at a 1:1 ratio, using Lipofectamine 2000. After a 24 h culture, the cells were treated with DMSO or compounds for 1 h. Nano-Glo Live Cell Reagent (Promega) was added, and luminescence was measured on a Nivo plate reader (PerkinElmer).

**TR-FRET assay**

GST-tagged human HOIL-1L and GST-tagged human SHARPIN were mixed with linear di-ubiquitin and tetra-ubiquitin chains, respectively, in FRET buffer containing 20 mM Tris-HCl pH 7.5, 0.1% BSA, and 0.01% Triton. After the addition of MAb anti 6His-d2 (Cisbio Bioassays) and MAb anti GST-Tb cryptate (Cisbio Bioassays), the samples were incubated for 1 h at room temperature. Fluorescence was measured using a Nivo plate reader (PerkinElmer).

**Pull-down assay**

MBP-His and MBP-His-tagged proteins were incubated with tetra-ubiquitin chains in incubation buffer containing 20 mM Tris-HCl pH 7.5, 150 mM NaCl, 20 mM ZnCl_2_, 10 mM imidazole, 1% Triton X-100, and 1 mM DTT for 1 h at 4°C with rotation. After adding 40 μL of Ni-NTA Agarose (QIAGEN), the samples were incubated for 1 h at 4°C with rotation. The beads were then washed three times with the incubation buffer and once with PBS. Proteins were eluted with 2× sample buffer, boiled at 95°C for 5 min, and analyzed by immunoblotting.

**Measurement of LDH release**

Cells were seeded at a density of 2 × 10^4^ cells per well in a 96-well plate and treated with TNF-α (2.5 ng/mL) plus CHX (20 μg/mL) or TNF-α (2.5 ng/mL) plus SM-164 (200 nM). After culture for the indicated periods, cell supernatants were analyzed for LDH activity using a CytoTox 96 Non-Radioactive Cytotoxicity Assay (Promega). Absorbance at 490 nm was measured using a SpectraMax M5 instrument (Molecular Devices).

**In vitro deubiquitination assay**

Linear di-ubiquitin (1 μM) was incubated with 1 μM USP2cc or 100 nM OTULIN in 20 μL DUB buffer (50 mM Tris-HCl pH 7.5, 50 mM NaCl, and 5 mM DTT) for 1 h at 37°C. Proteins were denatured by adding 4× sample buffer and boiling at 95°C for 5 min, and analyzed by SDS-PAGE.

**Blind docking simulation**

Blind docking simulations were performed using QuickVina-W [6], a variant of Autodock Vina, to predict the binding site and pose of the ligands simultaneously without prior knowledge of the binding site location. This approach was chosen because the binding sites of both HOIL-1L-NZF and SHARPIN-NZF were unknown. The initial structures of the ligands, FSL0717 and FSL0720, were prepared using MOE2022 [7]. The atomic models of the NZF domains of HOIL-1L and SHARPIN were derived from the crystal structure in complex with linear ubiquitin (PDB entry 3B08) and the predicted model in the AlphaFold Protein Structure Database [8] (accession ID Q91WA6), respectively. The grid boxes were defined with their centers as the centroids of the protein structures, ensuring that the boxes covered the entire surfaces. During the docking process, 20 output poses per molecule were generated and docked over the surface of the proteins with an exhaustiveness of 64. The models with the lowest docking scores were used to investigate the interactions.

**Statistical analysis**

Data are shown as the mean ± SD. Statistical significance was determined by one-sample t-test, two-tailed Student’s t test, or ANOVA with Tukey’s post hoc test. Data visualization and statistical analyses were performed using GraphPad Prism 9 (version 9.5.1). All experiments were performed at least twice, and consistent results were obtained.

**Supplementary references**

1. Tokunaga F, Sakata S, Saeki Y, Satomi Y, Kirisako T, Kamei K, et al. Involvement of linear polyubiquitylation of NEMO in NF-kappaB activation. Nat Cell Biol. 2009;11:123-32.

2. Tokunaga F, Nakagawa T, Nakahara M, Saeki Y, Taniguchi M, Sakata S, et al. SHARPIN is a component of the NF-κB-activating linear ubiquitin chain assembly complex. Nature. 2011;471:633-6.

3. Fujita H, Rahighi S, Akita M, Kato R, Sasaki Y, Wakatsuki S, et al. Mechanism underlying IκB kinase activation mediated by the linear ubiquitin chain assembly complex. Mol Cell Biol. 2014;34:1322-35.

4. Bochkov YA, Palmenberg AC. Translational efficiency of EMCV IRES in bicistronic vectors is dependent upon IRES sequence and gene location. Biotechniques. 2006;41:283-4, 6, 8 passim.

5. Fujita H, Tokunaga A, Shimizu S, Whiting AL, Aguilar-Alonso F, Takagi K, et al. Cooperative Domain Formation by Homologous Motifs in HOIL-1L and SHARPIN Plays A Crucial Role in LUBAC Stabilization. Cell Rep. 2018;23:1192-204.

6. Hassan NM, Alhossary AA, Mu Y, Kwoh CK. Protein-Ligand Blind Docking Using QuickVina-W With Inter-Process Spatio-Temporal Integration. Sci Rep. 2017;7:15451.

7. Molecular Operating Environment (MOE), 2022.02 Chemical Computing Group ULC, 910-1010 Sherbrooke St. W., Montreal, QC H3A 2R7, Canada, 2024.

8. Varadi M, Anyango S, Deshpande M, Nair S, Natassia C, Yordanova G, et al. AlphaFold Protein Structure Database: massively expanding the structural coverage of protein-sequence space with high-accuracy models. Nucleic Acids Res. 2022;50:D439-D444.


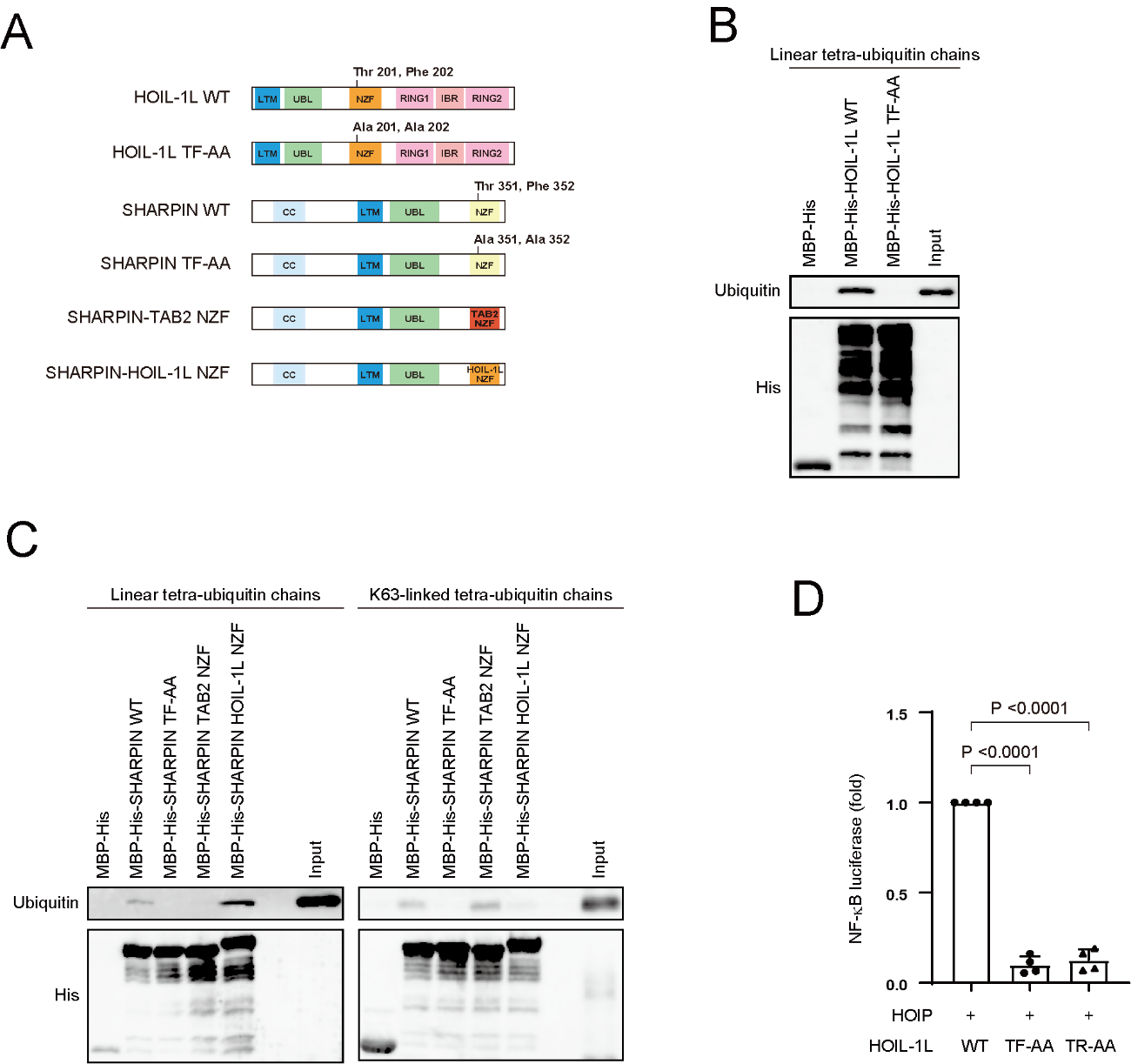


**Supplementary Fig. 1 The TF-AA mutants of HOIL-1L and SHARPIN are unable to bind to ubiquitin chains.**

**A** Schematic illustration of WT and mutant forms of HOIL-1L and SHARPIN. **B, C** MBP-His, MBP-His-tagged HOIL-1L (**B**), and MBP-His-tagged SHARPIN (**C**) were incubated with linear tetra-ubiquitin chains or K63-linked tetra-ubiquitin chains for 1 h and pulled down by Ni-NTA Agarose. Samples were analyzed by immunoblotting with the indicated antibodies. Data are representative of three independent experiments. **D** NF-κB activation in HEK293T cells transiently transfected with the indicated combinations of HOIP and HOIL-1L was measured using a luciferase assay. Data are shown as the mean ± s.d. of four independent experiments. P values were obtained using a one-sample t test. Uncropped western blots are available for this figure in the Supplementary Material.


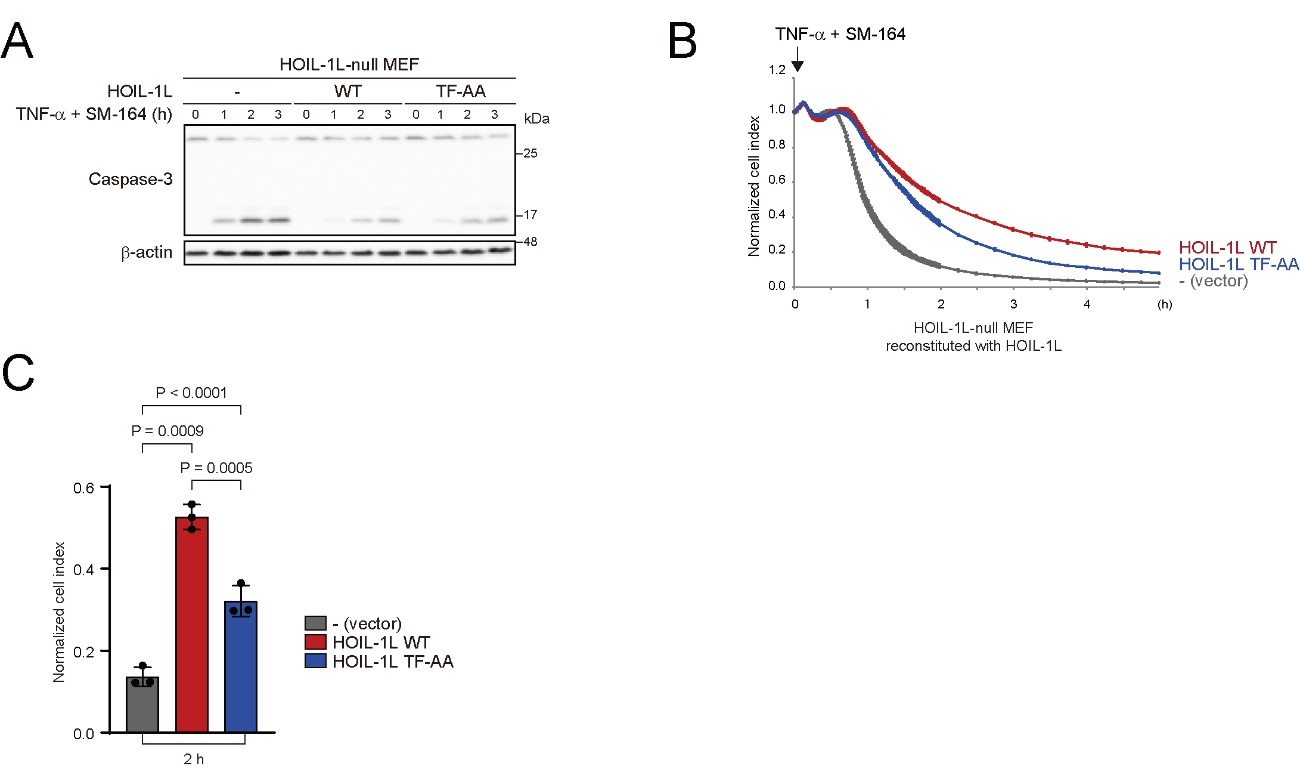


**Supplementary Fig. 2 Cell death analyses of reconstituted HOIL-1L-null MEFs stimulated with TNF-α plus SM-164**

**A** HOIL-1L-null MEFs stably reconstituted with the indicated proteins were stimulated with TNF-α (2.5 ng/mL) plus SM-164 (200 nM) for the indicated times, and analyzed by immunoblotting with the indicated antibodies. Data are representative of three independent experiments. **B, C** HOIL-1L-null MEFs stably reconstituted with the indicated proteins were stimulated with TNF-α (2.5 ng/mL) and SM-164 (200 nM), and cell viability was continuously measured using the xCELLigence system. A representative image (**B**) and statistical analyses of the normalized cell index at 2 h (**C**). Data are shown as the mean ± s.d. of three independent experiments. P values were calculated by a one-way ANOVA with Tukey’s post hoc test. Uncropped western blots are available for this figure in the Supplementary Material.


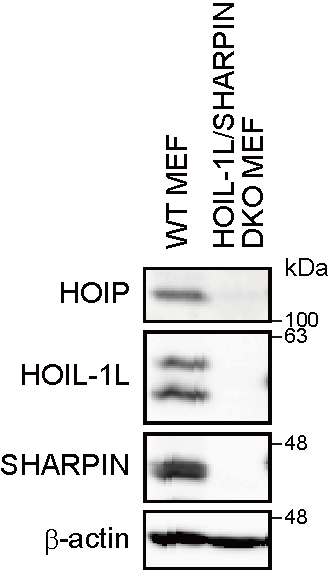


**Supplementary Fig. 3 Confirmation of HOIL-1L/SHARPIN double knockout in MEFs.**

Immunoblot analyses of lysates of WT MEFs and HOIL-1L/SHARPIN DKO MEFs. Data are representative of three independent experiments. Uncropped western blots are available for this figure in the Supplementary Material.


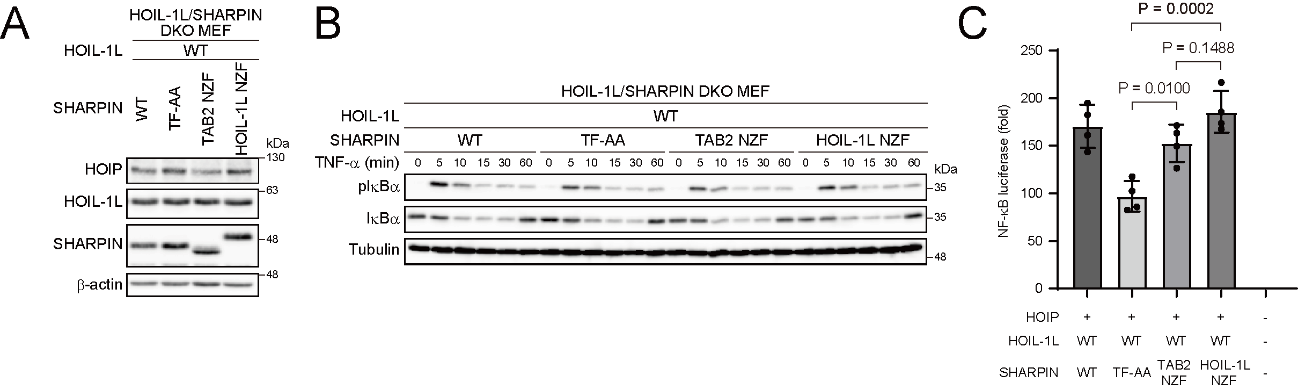


**Supplementary Fig. 4 SHARPIN NZF recognition of linear and K63-linked ubiquitin chains contributes to NF-κB activation in the presence of HOIL-1L NZF WT.**

**A** Immunoblot analyses of lysates of HOIL-1L/SHARPIN DKO MEFs stably reconstituted with the indicated proteins. Data are representative of three independent experiments. **B** HOIL-1L/SHARPIN DKO MEFs stably reconstituted with the indicated proteins were stimulated with TNF-α (5 ng/mL) for the indicated times and analyzed by immunoblotting with the indicated antibodies. Data are representative of three independent experiments. **C** NF-κB activation in HEK293T cells transiently transfected with the indicated combinations of HOIP, HOIL-1L, and SHARPIN was measured using a luciferase assay. Data are shown as the mean ± s.d. of four independent experiments. P values are from one-way ANOVA with Tukey’s post hoc test. Uncropped western blots are available for this figure in the Supplementary Material.


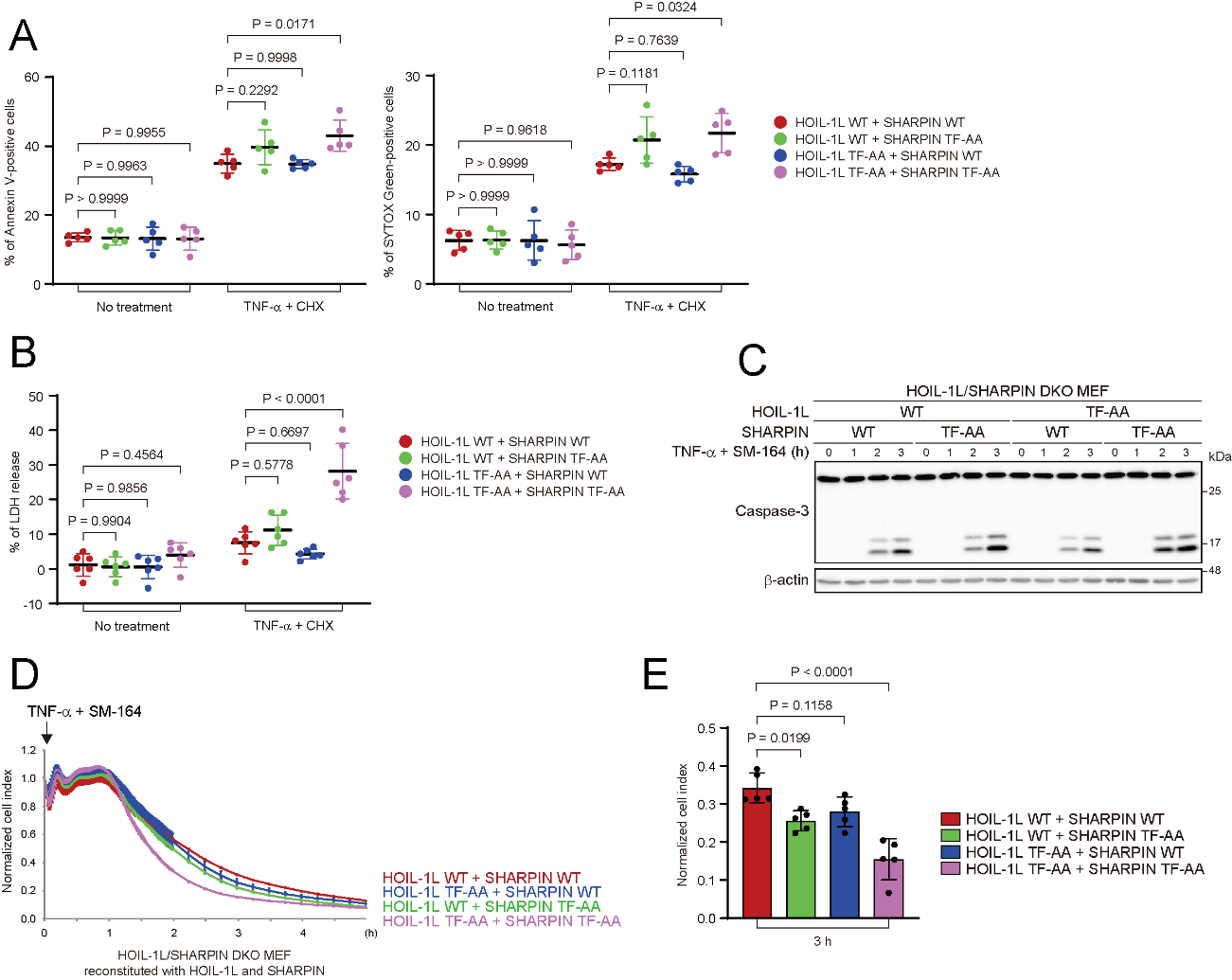


**Supplementary Fig. 5 Cell death analyses of reconstituted DKO MEFs stimulated with TNF-α plus CHX or TNF-α plus SM-164.**

**A** HOIL-1L/SHARPIN DKO MEFs stably reconstituted with the indicated proteins were stimulated with TNF-α (2.5 ng/mL) plus CHX (20 μg/mL) for 0 or 8 h. Samples were analyzed by flow cytometry after Annexin V and SYTOX Green staining. Data are displayed as the frequencies of Annexin V-positive cells (left) and SYTOX Green-positive cells (right). Data are shown as the mean ± s.d. of five independent experiments. P values are from a one-way ANOVA with Tukey’s post hoc test. **B** HOIL-1L/SHARPIN DKO MEFs stably reconstituted with the indicated proteins were stimulated with TNF-α (2.5 ng/mL) plus CHX (20 μg/mL) for 0 or 6 h, and cell death was measured using a LDH activity assay. Data are shown as the mean ± s.d. of six independent experiments. P values are from a one-way ANOVA with Tukey’s post hoc test. **C** HOIL-1L/SHARPIN DKO MEFs stably reconstituted with the indicated proteins were stimulated with TNF-α (2.5 ng/mL) plus SM-164 (200 nM) for the indicated times, and analyzed by immunoblotting with the indicated antibodies. Data are representative of three independent experiments. **D, E** HOIL-1L/SHARPIN DKO MEFs stably reconstituted with the indicated proteins were stimulated with TNF-α (2.5 ng/mL) and SM-164 (200 nM), and cell viability was continuously measured using the xCELLigence system. A representative image (**D**) and statistical analyses of normalized cell index at 3 h (**E**). Data are shown as the mean ± s.d. of five independent experiments. P values were calculated by a one-way ANOVA with Tukey’s post hoc test. Uncropped western blots are available for this figure in the Supplementary Material.


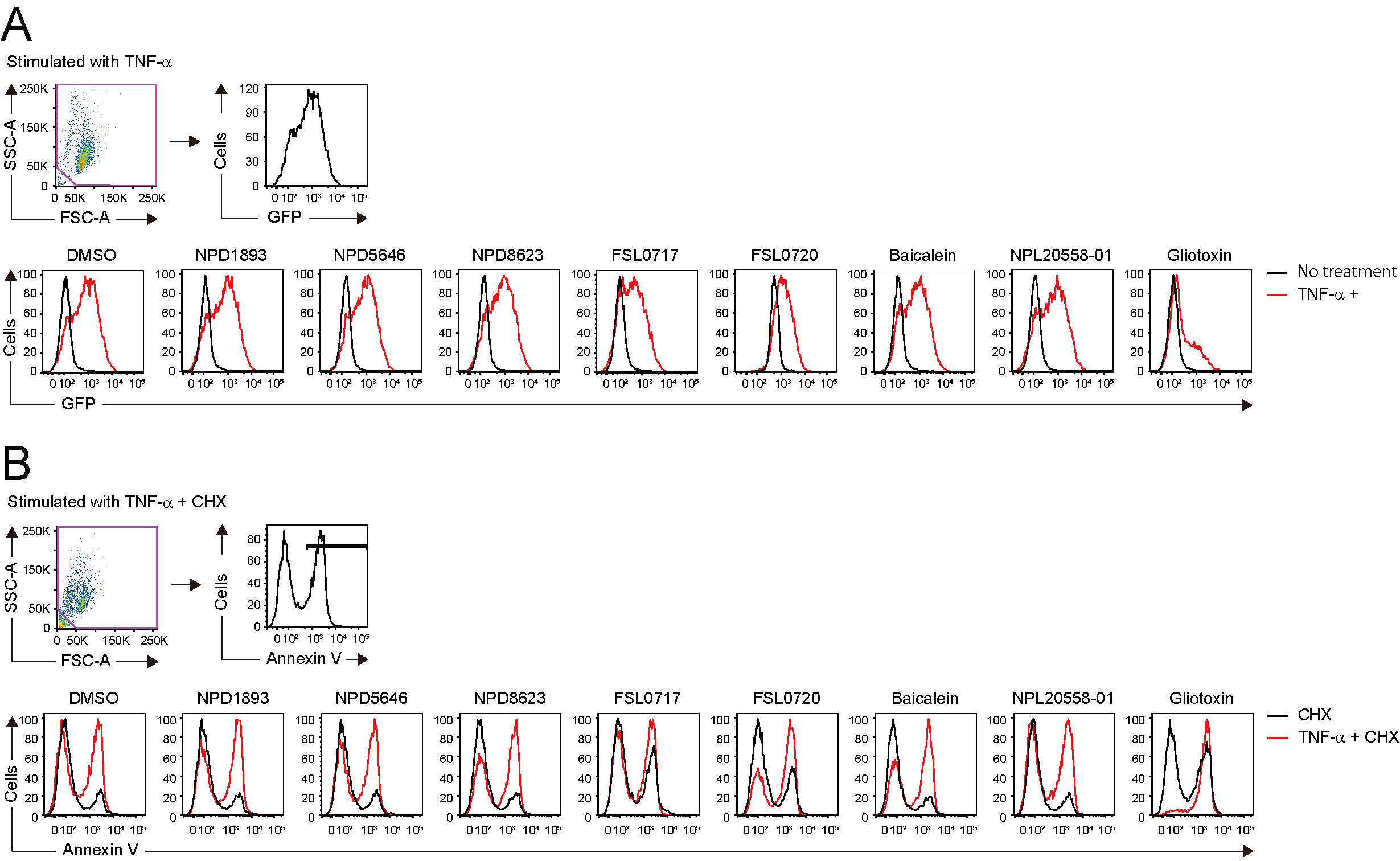


**Supplementary Fig. 6 Flow cytometry analysis of JR-GFP cells stimulated with TNF-α or TNF-α plus CHX after treatment with compounds.**

**A, B** Gating strategy for the flow cytometry analysis shown in Figure 4C and D and representative images. Live cells were gated with forward-scatter (FSC) and side-scatter (SSC), and the mean fluorescence intensity (MFI) of GFP expression (**A**) and the frequency of Annexin V-positive cells (**B**) were calculated. The black bar represents the range of Annexin V-positive cells (**B**).


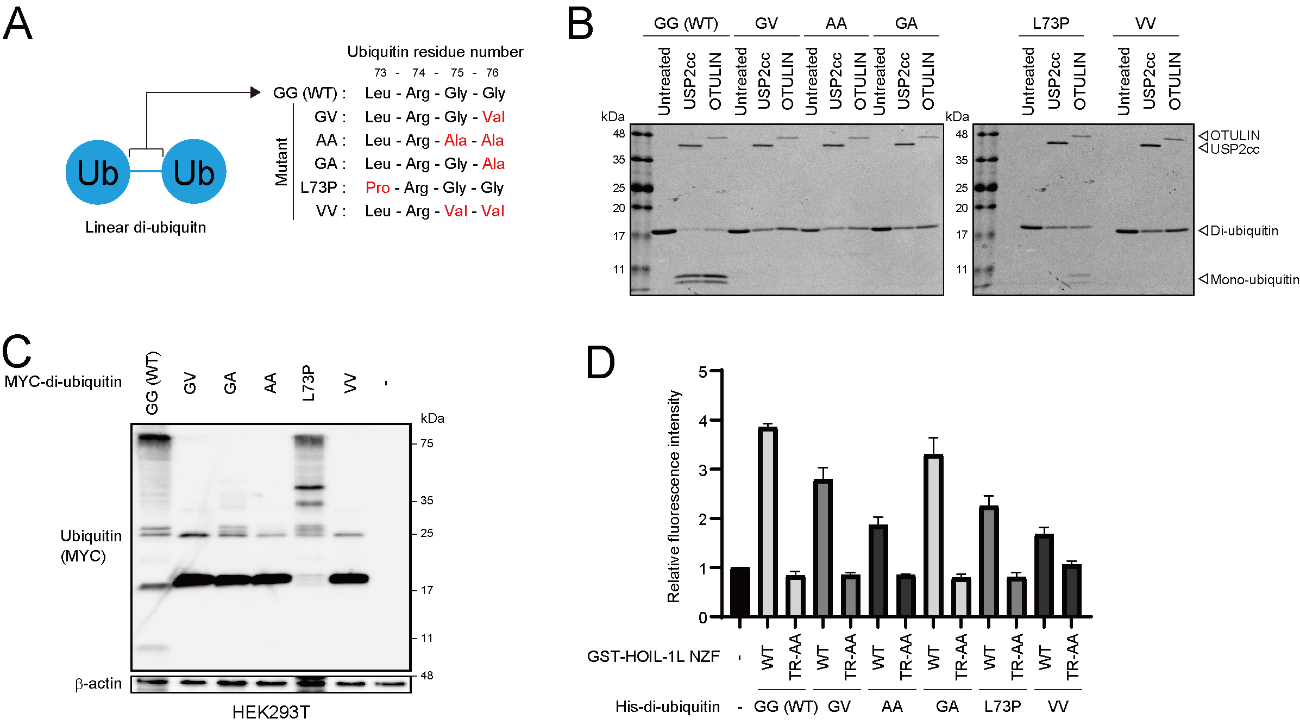


**Supplementary Fig. 7 Investigation of DUB-resistant linear ubiquitin chains.**

**A** Schematic illustration of WT and mutant linear di-ubiquitin. **B** WT or mutant forms of linear di-ubiquitin were incubated with USP2cc or OTULIN for 1 h at 37°C and analyzed using SDS-PAGE followed by Coomassie brilliant blue staining. Data are representative of two independent experiments. **C** HEK293T cells were transfected with MYC-tagged linear di-ubiquitin for 48 h and analyzed by immunoblotting with the indicated antibodies. Data are representative of two independent experiments. **D** His-tagged di-ubiquitin and GST-tagged HOIL-1L NZF were mixed and incubated for 1 h. Fluorescence intensity was measured using a FRET assay. Data are shown as the mean ± s.d. of two independent experiments. Uncropped western blots are available for this figure in the Supplementary Material.


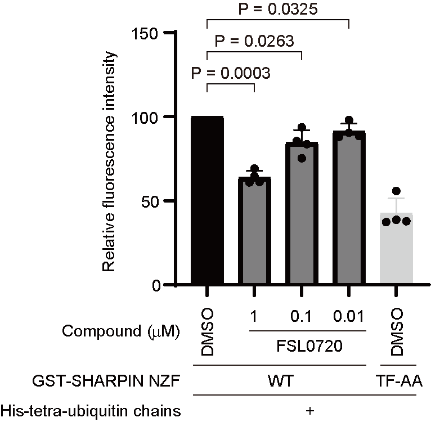


**Supplementary Fig. 8 FSL0720 inhibits the binding between SHARPIN NZF and linear ubiquitin chains in a dose-dependent manner.**

His-tagged di-ubiquitin and GST-tagged SHARPIN NZF were mixed with DMSO or FSL0720 and incubated for 1 h. Fluorescence intensity was measured using the FRET assay. Data are shown as the mean ± s.d. of four independent experiments. P values are from a one-sample t test.


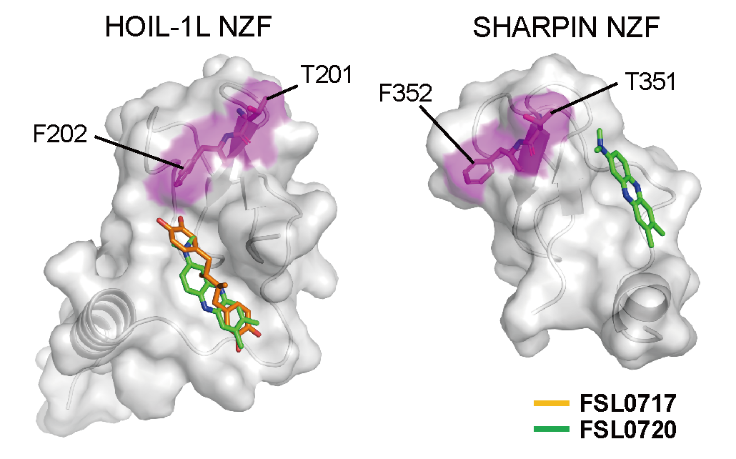


**Supplementary Fig. 9 The docking models of HOIL-1L NZF and SHARPIN NZF with compounds.**

The docking simulation for HOIL-1L NZF was conducted using the atomic model derived from the crystal structure (PDB entry 3b08) (left). For SHARPIN NZF, the docking simulation utilized the predicted structure from the AlphaFold Protein Structure Database (accession ID Q91WA6) because an experimental structure was unavailable (right). The models with the lowest docking scores are shown. FSL0717 and FSL0720 are represented by stick models and their carbon atoms are colored orange and green, respectively. The residues critical for recognizing ubiquitin chains are represented by stick models and highlighted in magenta.

**Supplementary Table** Screening of compounds for inhibitors of GST-tagged HOIL-1L NZF and His-tagged linear di-ubiquitin interactions by TR-FRET assay

|  | RIKEN NPDepo libraries | DDI core library | FDA approved drugs |
| --- | --- | --- | --- |
| Primary screen | 20,393 compounds | 9,600 compounds | 3,865 compounds |
| Final concentration of test compound in the TR-FRET assay mixture (20 µL) | 5 µg/mL or 5 µM | 5 µM | 2.5 µM |
| - GST-tagged HOIL-1L NZF : His-tagged linear di-ubiquitin TR-FRET assay (n=1) | 265 compounds (Inh%: ≥50) | 151 compounds (Inh%: ≥30) | 85 compounds (Inh%: ≥40) |
| Secondary screen | 252 compounds (provided by the Drug Discovery Chemical Bank Unit, RIKEN) | 1. compounds (provided by the DDI, the University of Tokyo) | 1. compounds (picked up from the library stock solution) |
| - Retest assay^a^ (n=4) | 212 compounds (Inh%: ≥50; criterion 1) | 119 compounds (Inh%: ≥50; criterion 1) | 60 compounds (Inh%: ≥40; criterion 1) |
| - Dose–response assay^b, c^ (n=2) | 191 compounds (IC50: ≤10 µM; criterion 2) | 119 compounds (IC50: ≤10µM; criterion 2) | 60 compounds (IC50: ≤10µM; criterion 2) |
| - Dose–response assay^b, c^ using a counter TR-FRET assay with 6His-tagged GST fusion protein (n=2) | 196 compounds (selected by criterion 3^d^) | 98 compounds (selected by criterion 3^d^) | 59 compounds (selected by criterion 3^d^) |
| Number of compounds that met criteria 1, 2, and 3 | 180 compounds | 98 compounds | 59 compounds |
| Hit compounds | 180 compounds | 86 compounds^e^ | 54 compounds^f^ |

^a^ Retest assay was performed at the same test compound concentrations as the primary screen.

^b^ For the secondary screen of the RIKEN NPDepo library, a dose-response test was performed with eight concentrations in 4-fold dilution series ranging from 10 μM to 0.61 nM for 36 test compounds in the TR-FRET assay mixture (20 μL). The remaining 216 test compounds were assessed from 10 μg/mL to 0.61 ng/mL and their IC50 values were converted into molar concentrations.

^c^ For the secondary screen of the DDI core and known drug library, the dose-response test was performed with eight concentrations in 4-fold dilution series ranging from 10 µM to 0.61 nM of test compound in the TR-FRET assay mixture (20 μL).

^d^ Criterion 3 is either counter_IC50: > 10 μM or ratio of IC50 (=Counter TR-FRET assay/ GST-tagged HOIL-1L NZF : His-tagged linear di-ubiquitin TR-FRET assay): ≥ 3.

^e^ Twelve compounds were excluded as frequent hitters according to a database of DDI.

^f^ The law-regulated five compounds were excluded.


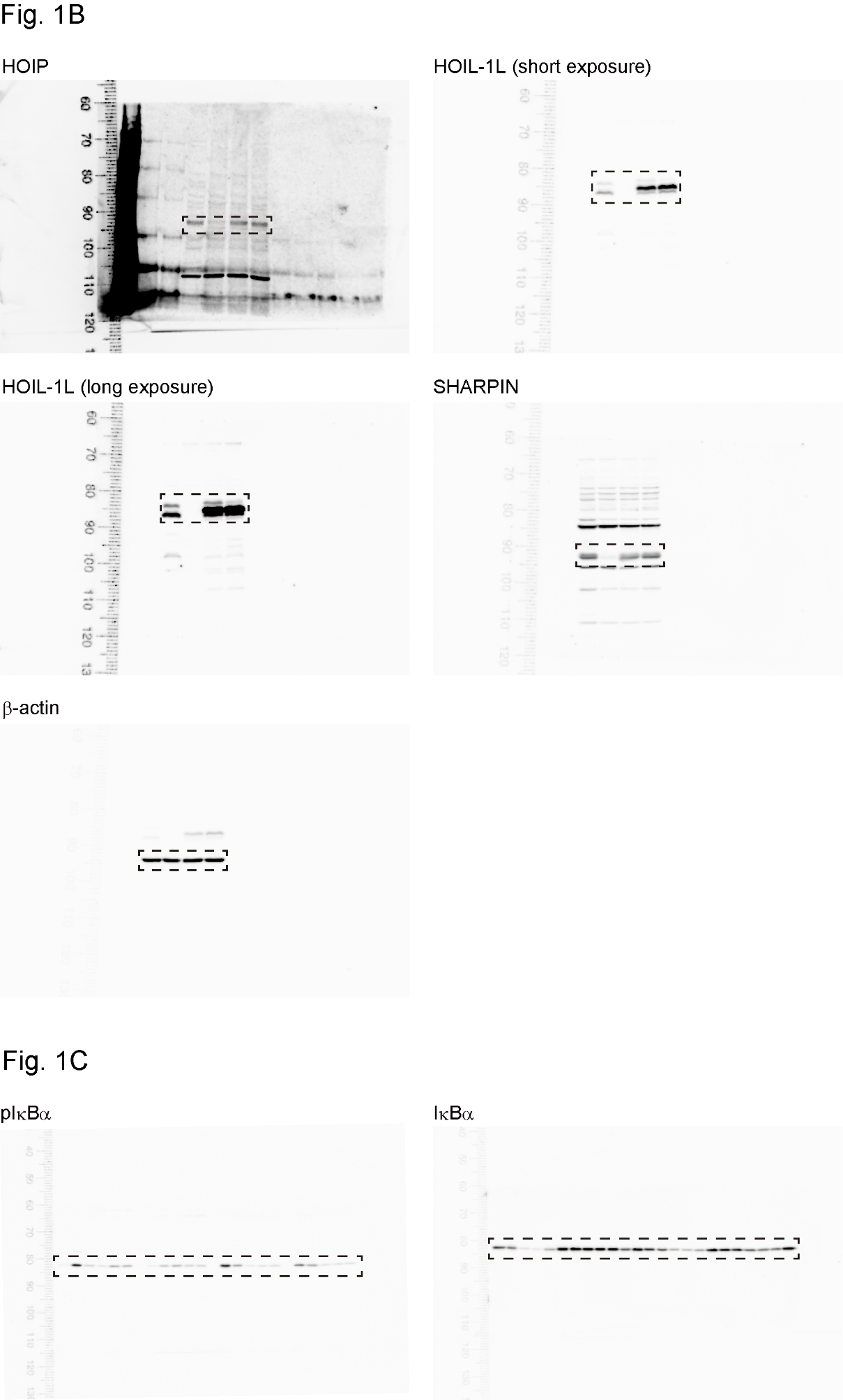


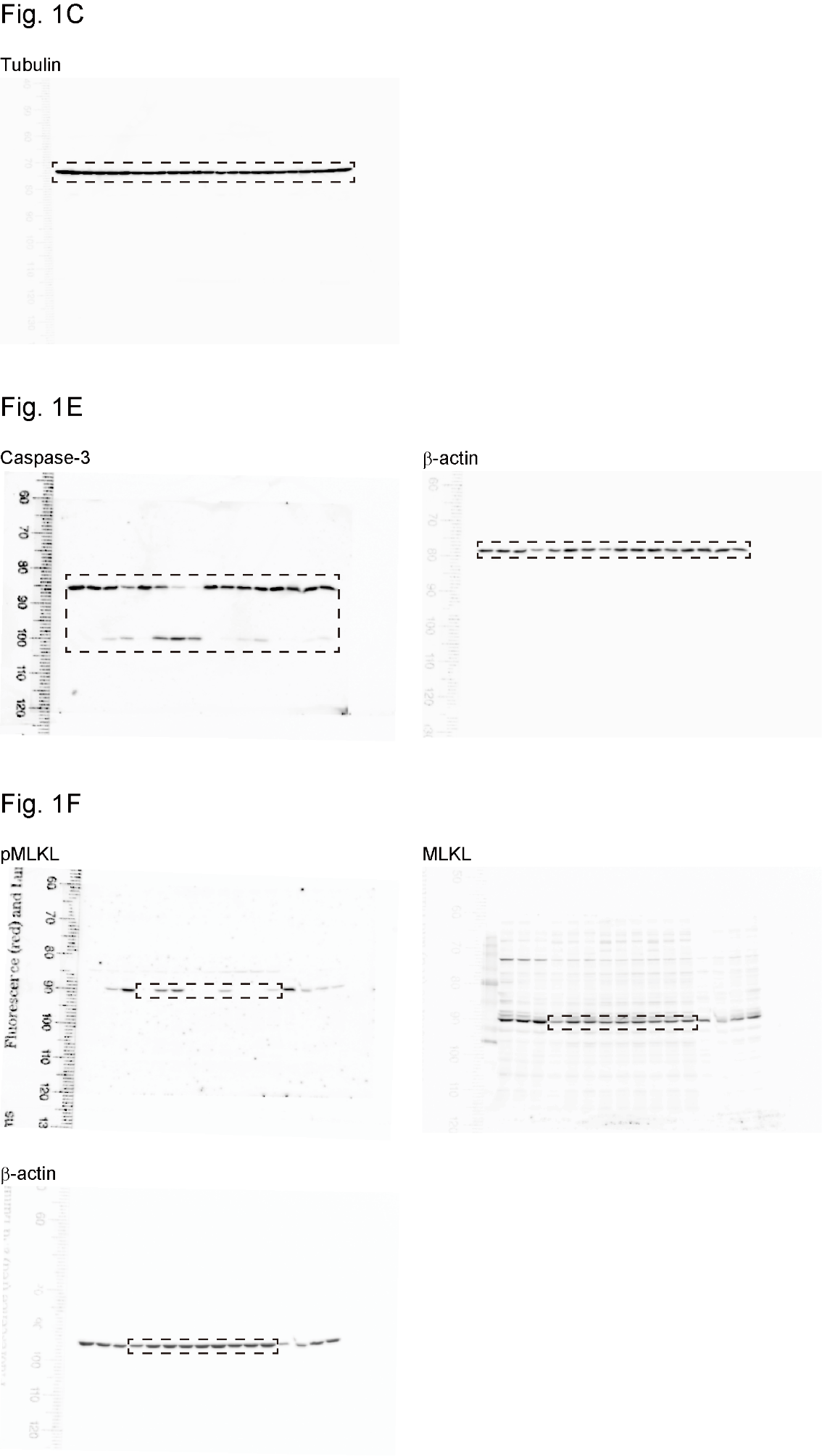


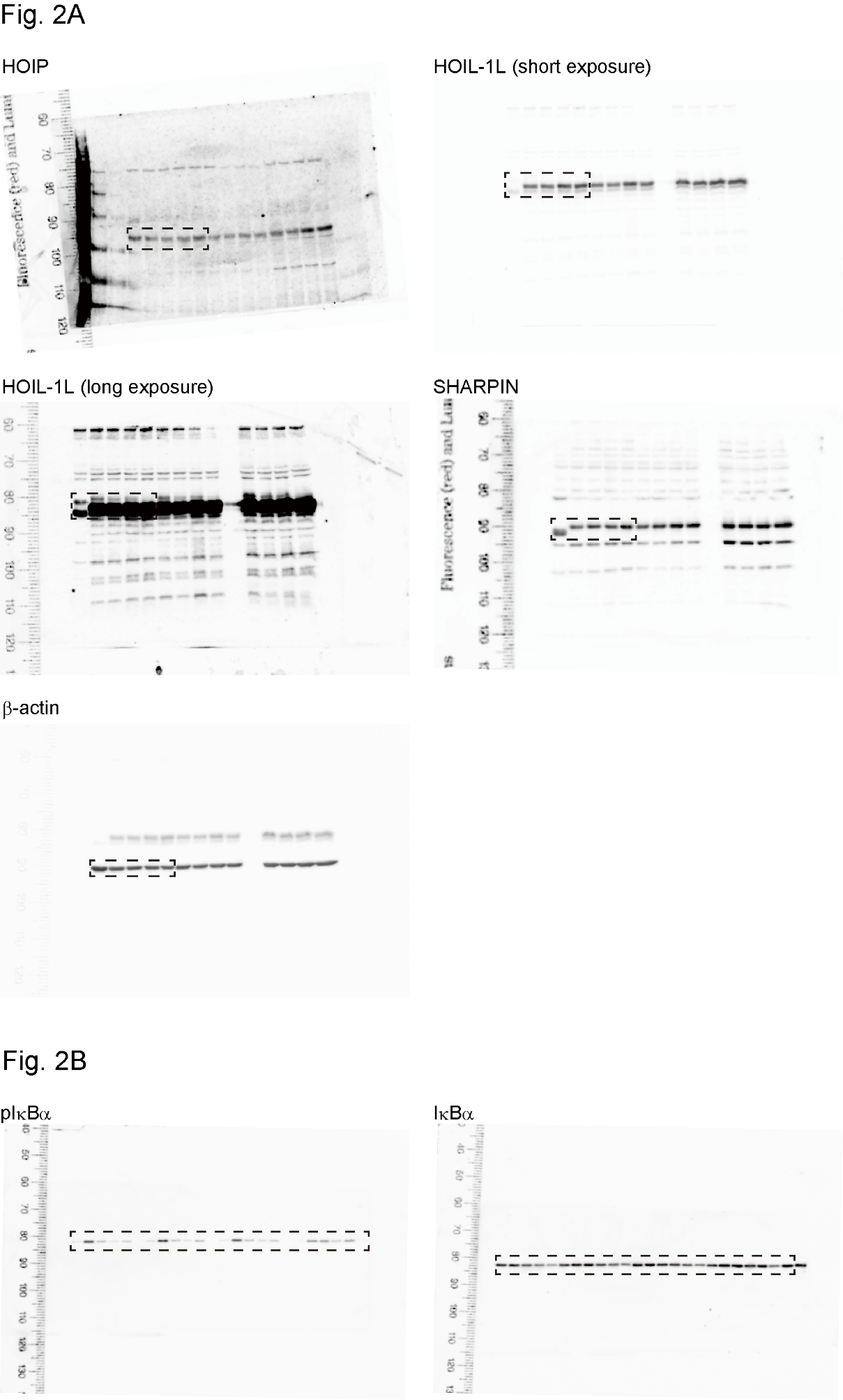


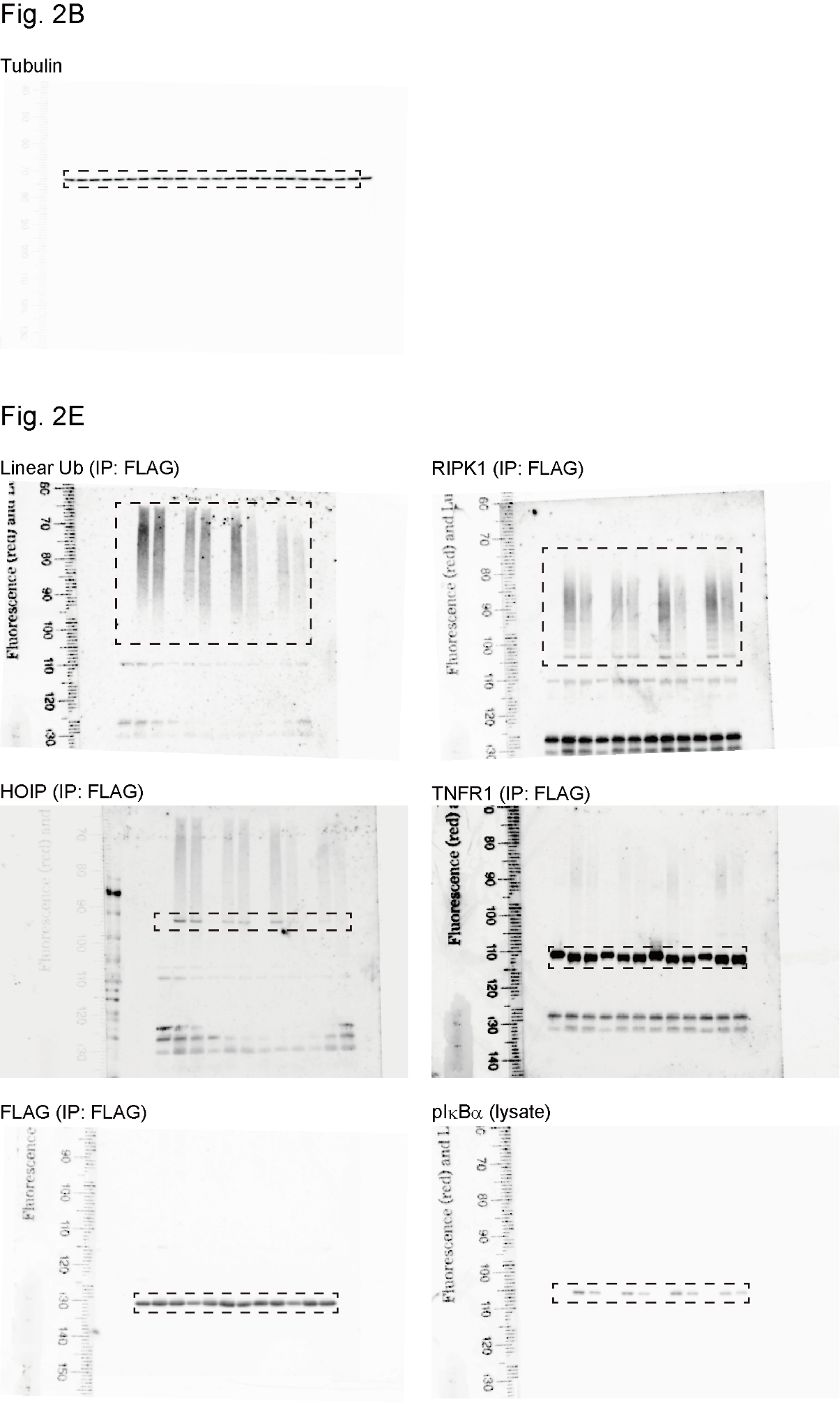


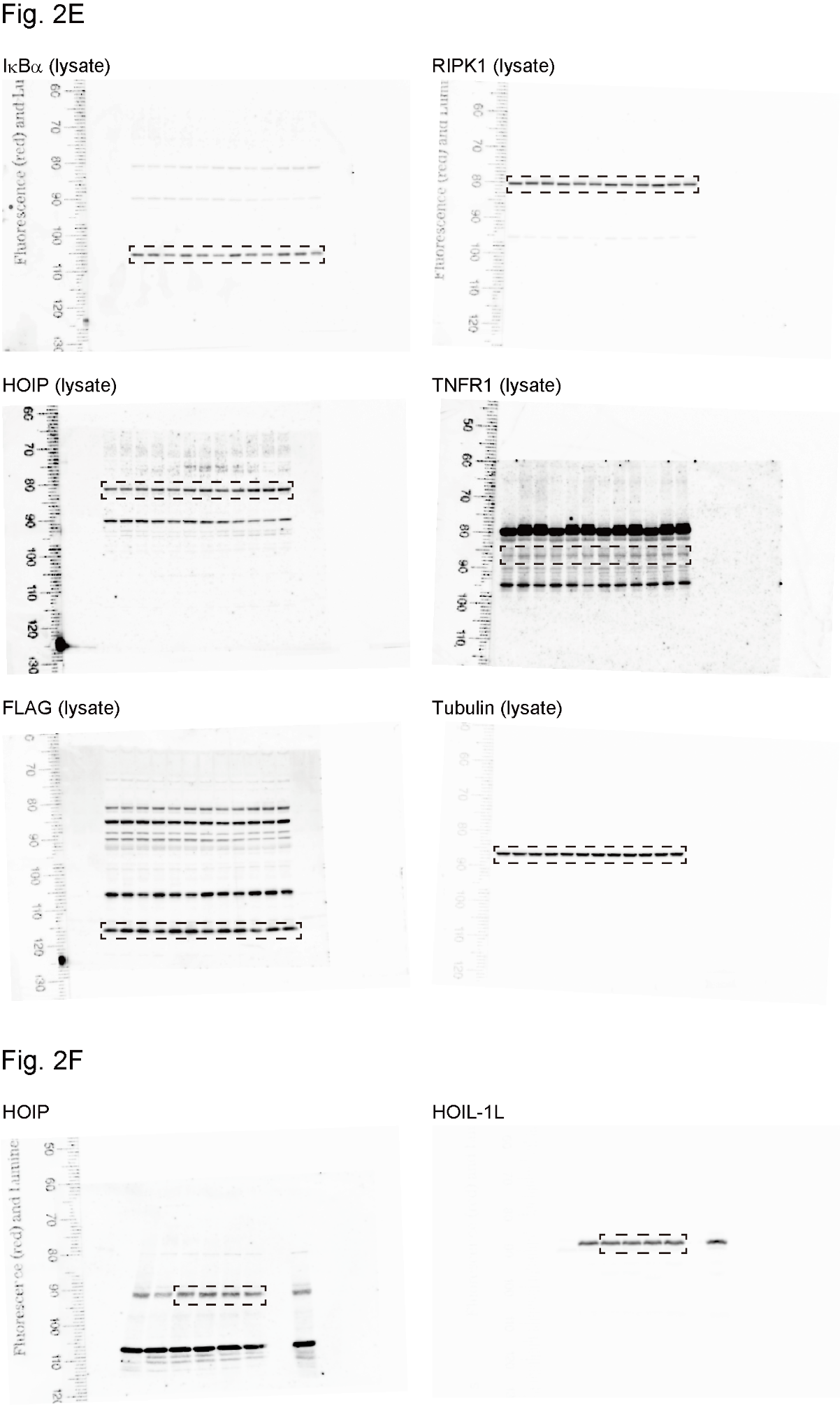


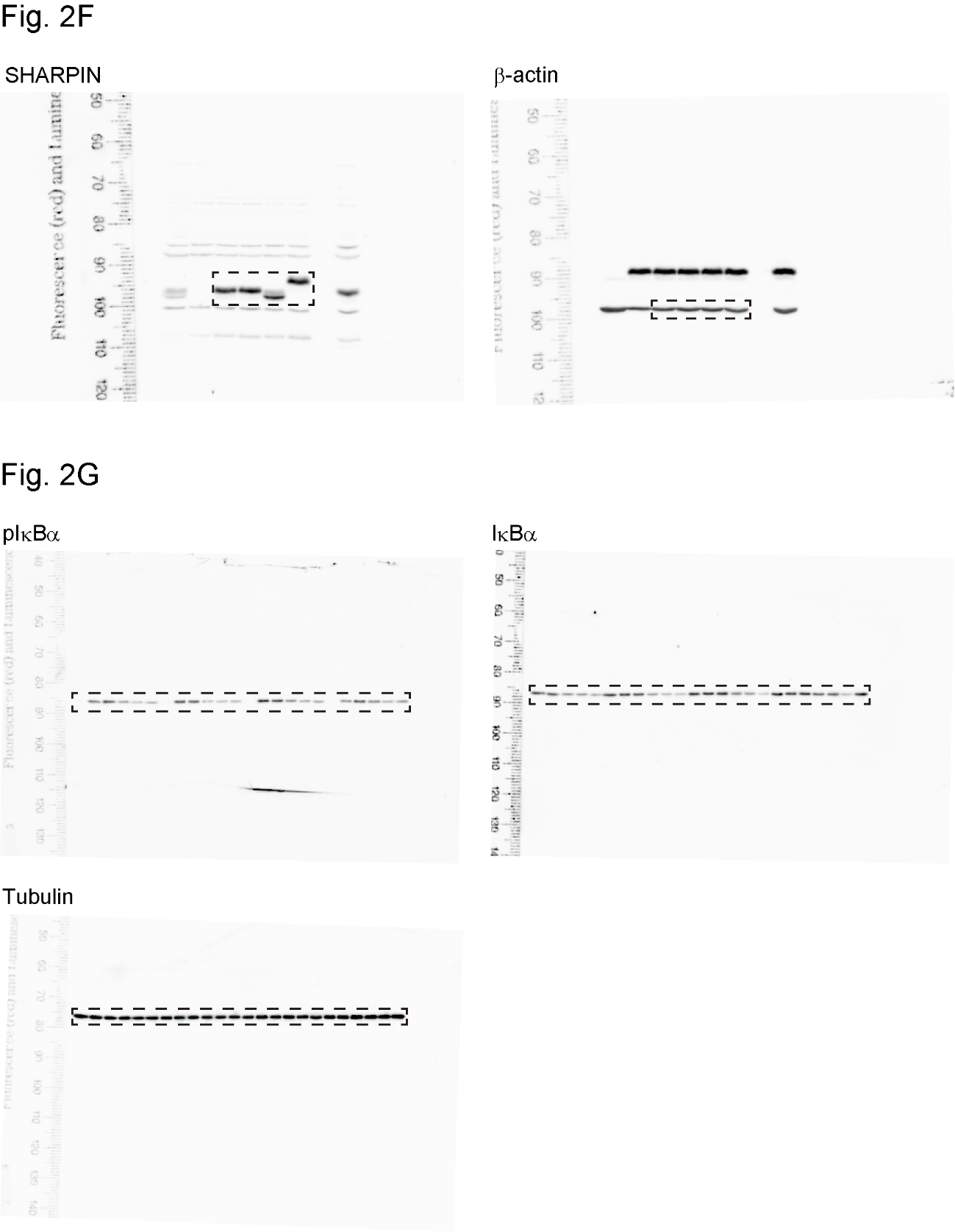


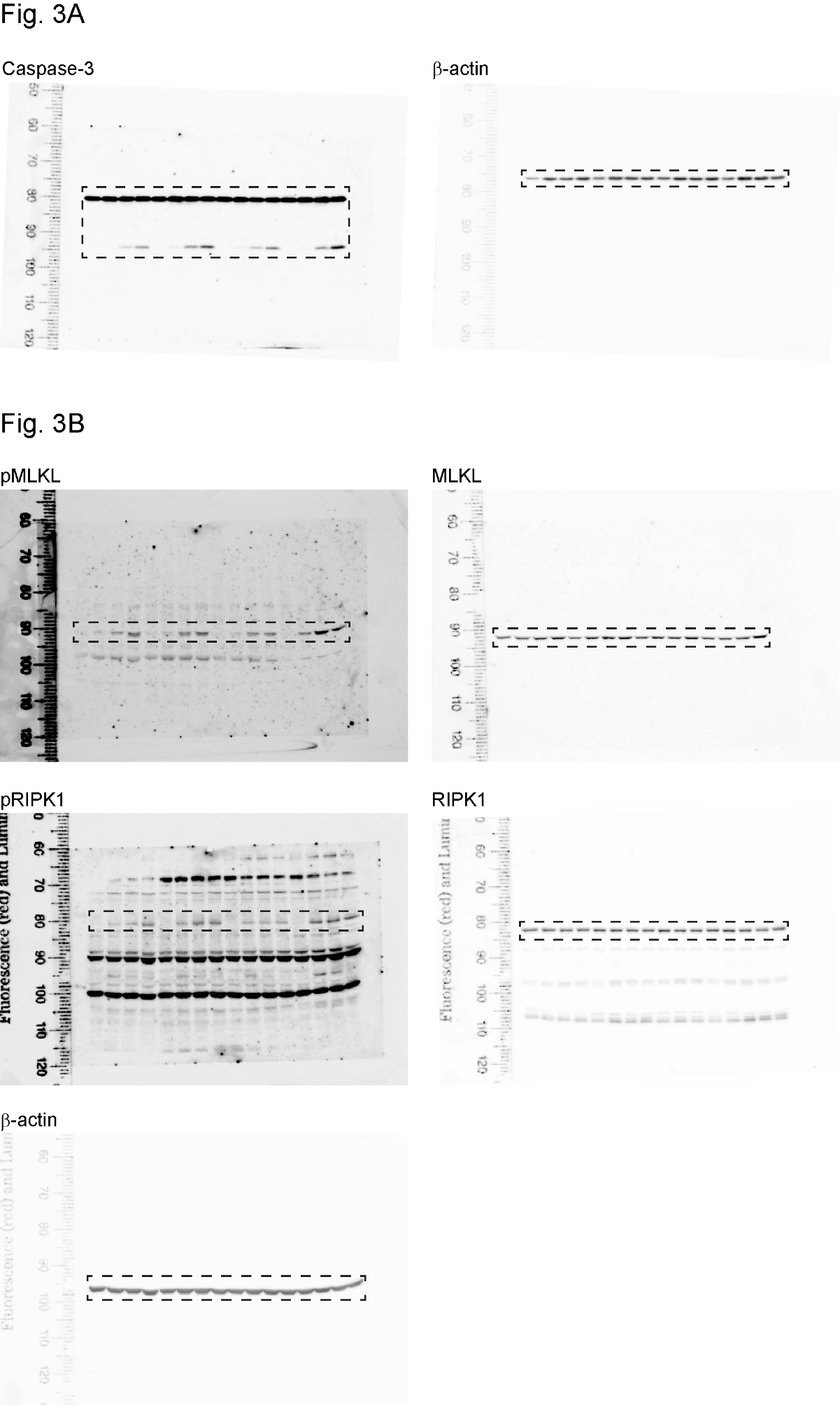


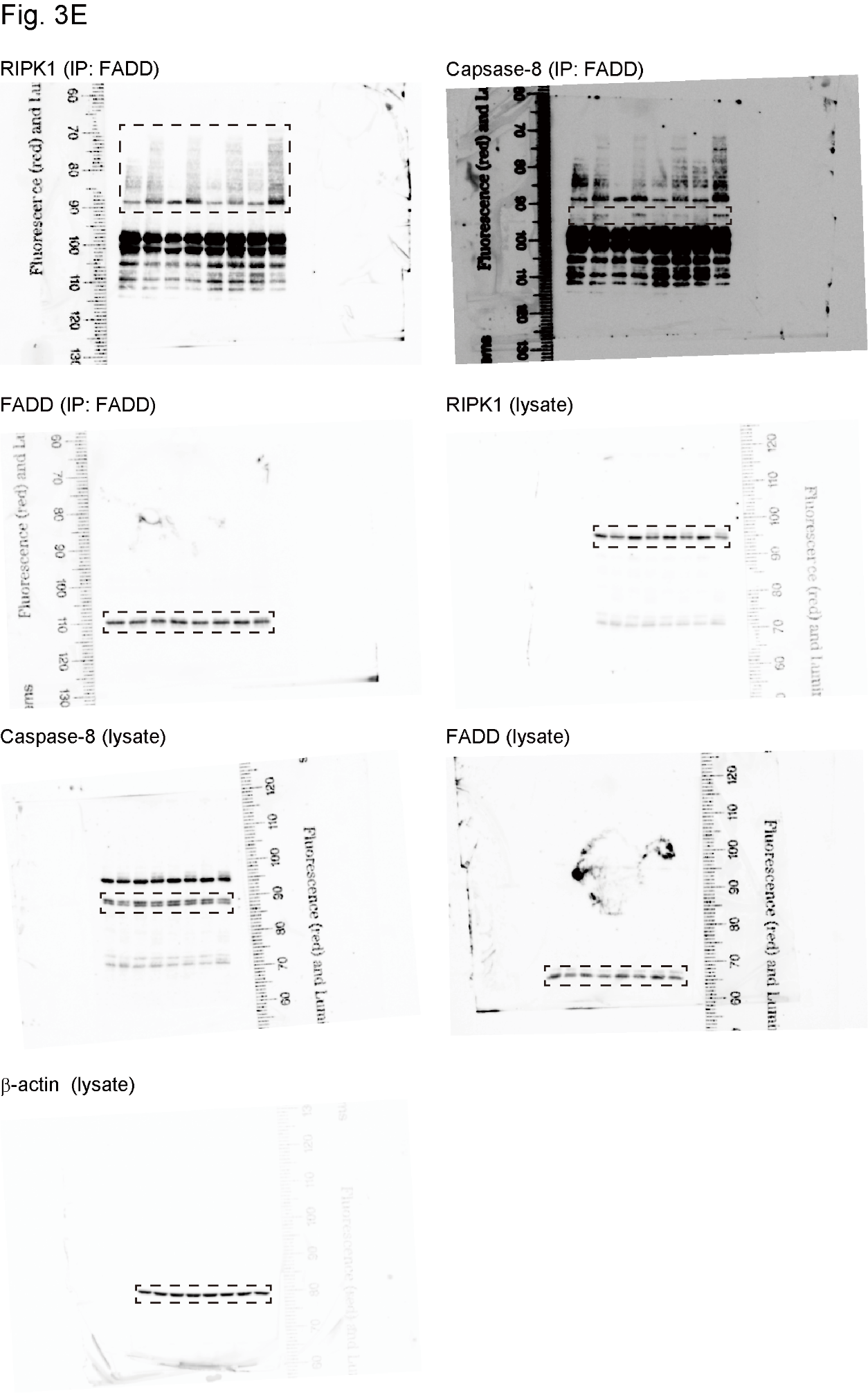


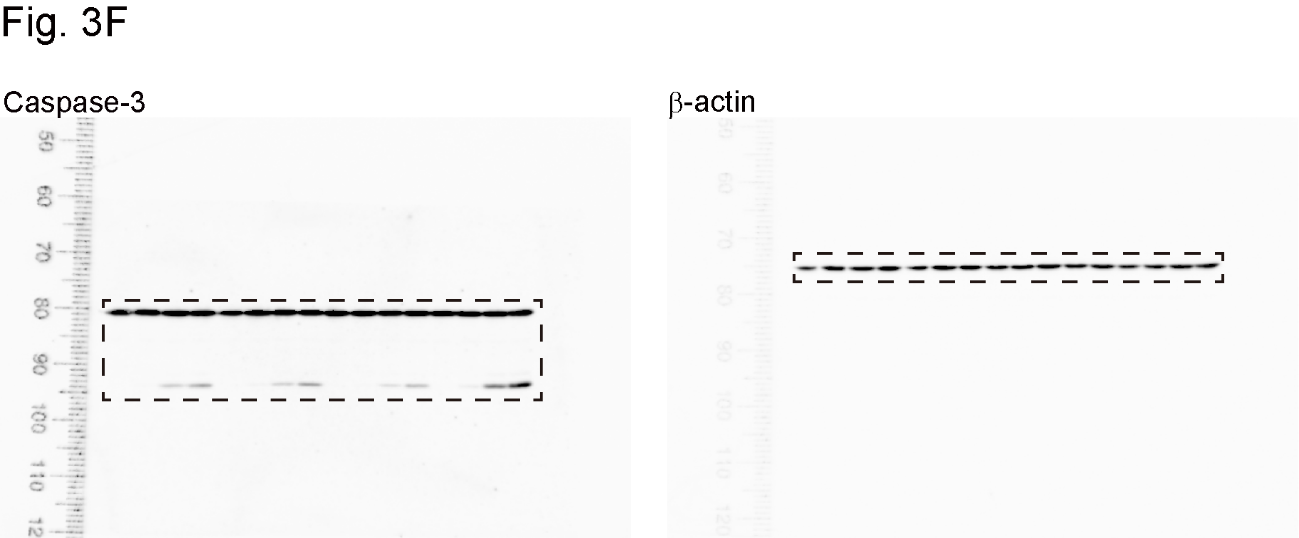


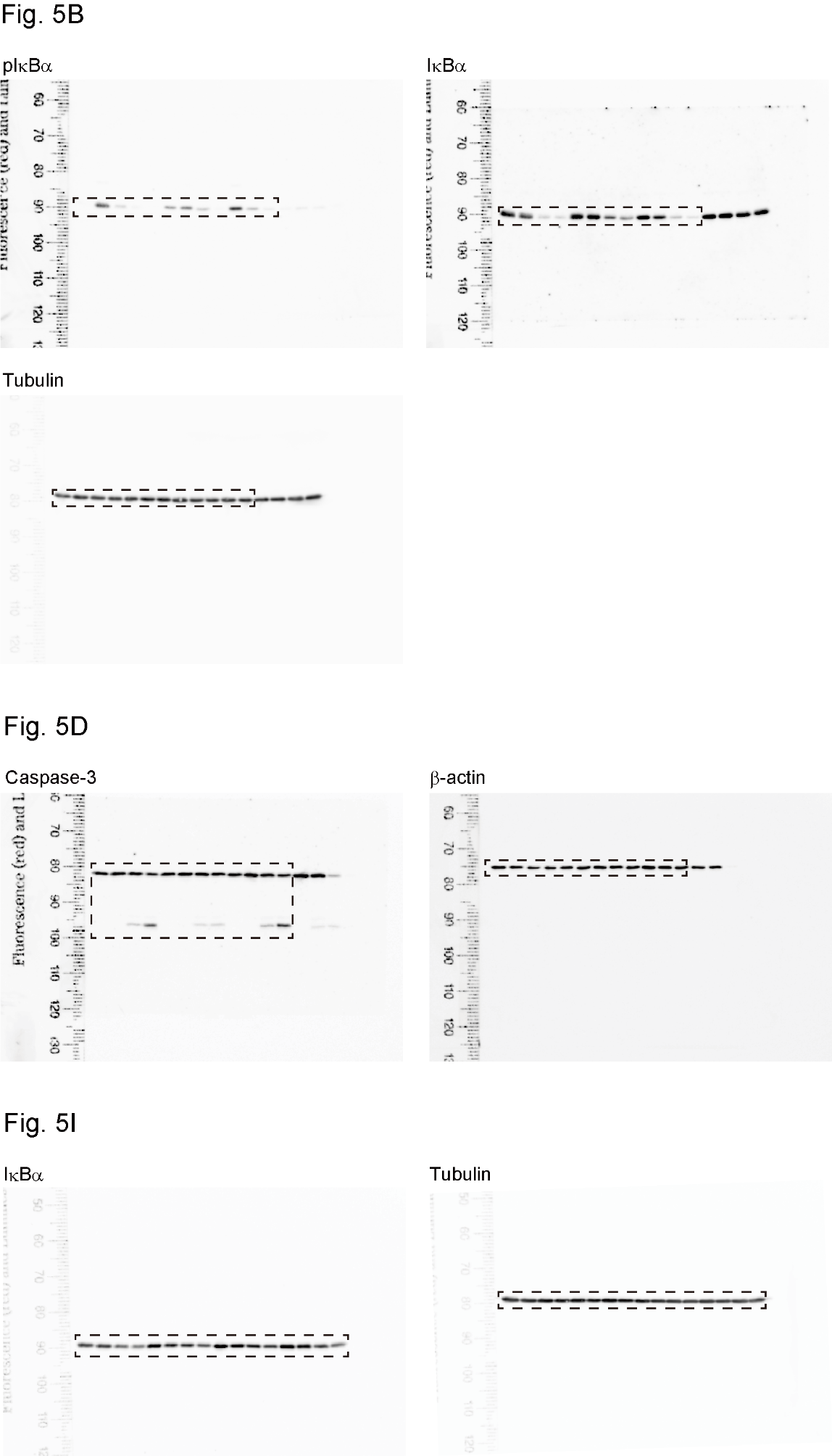


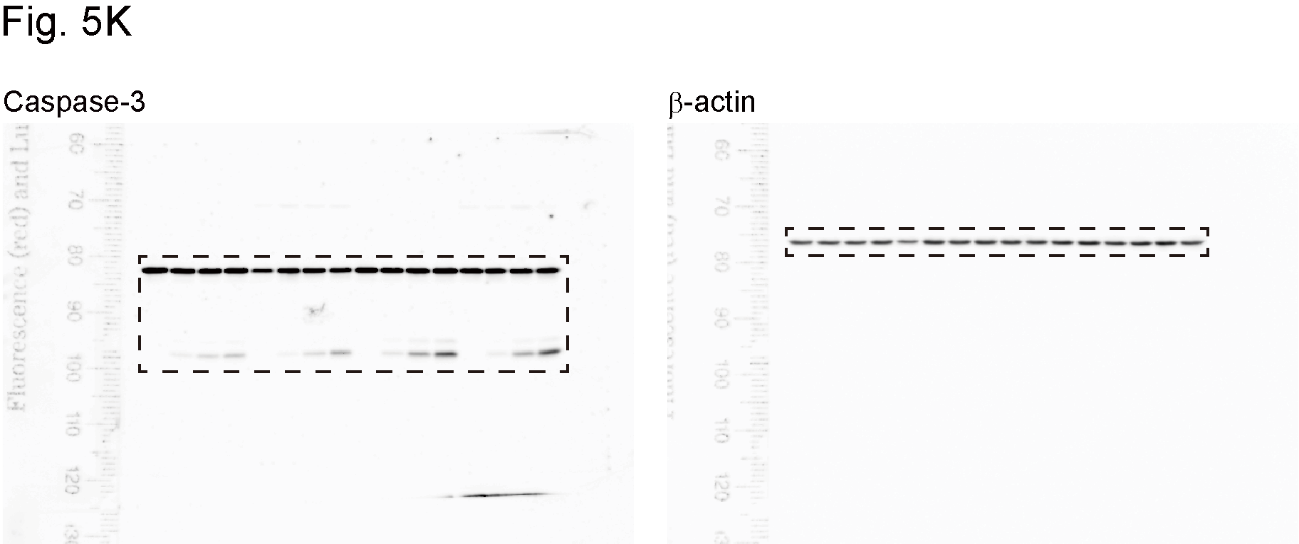


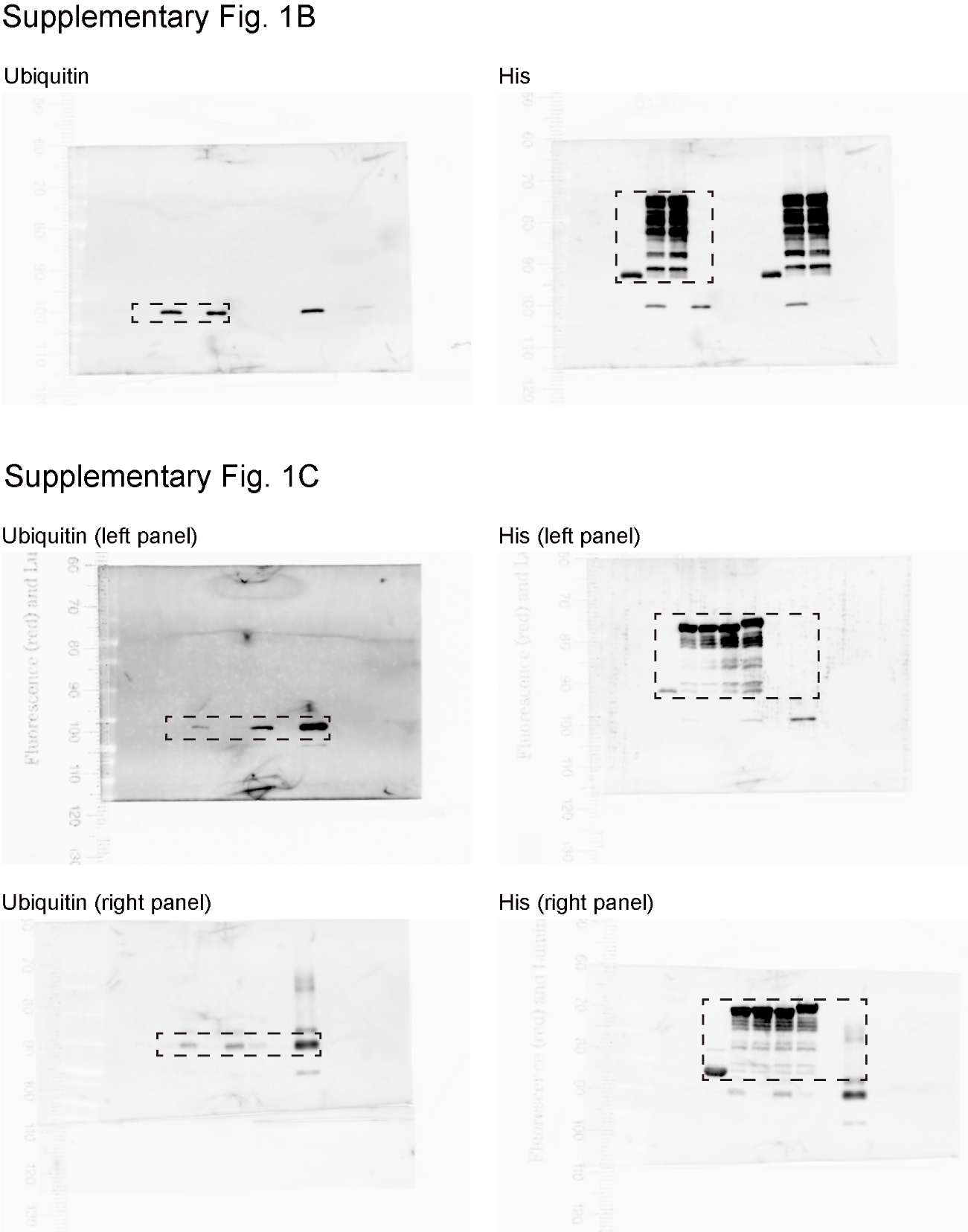


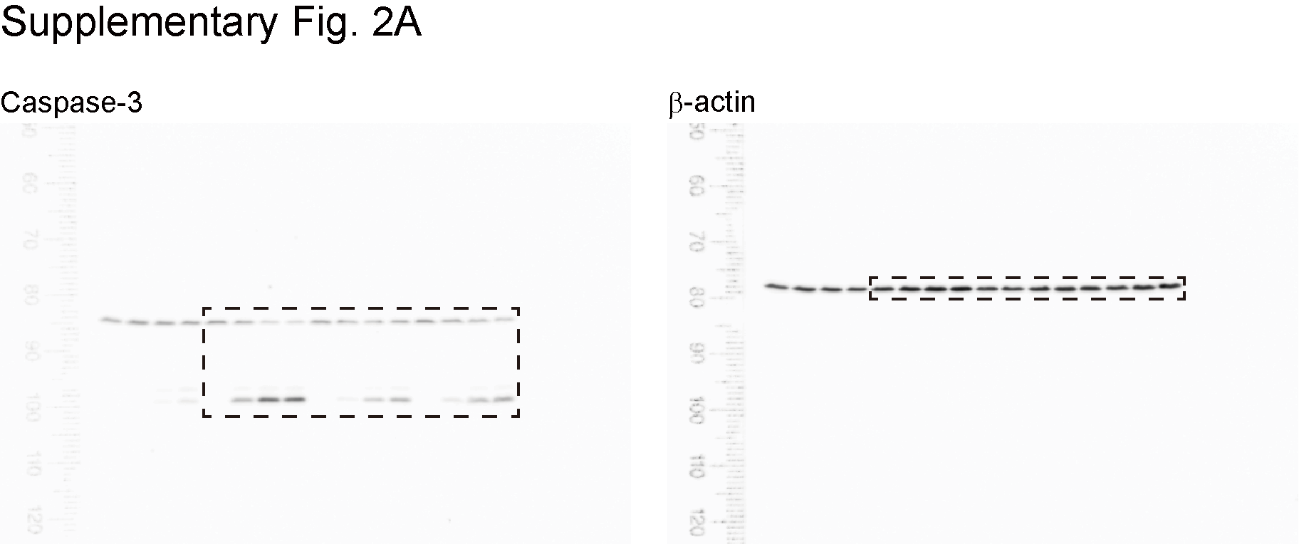


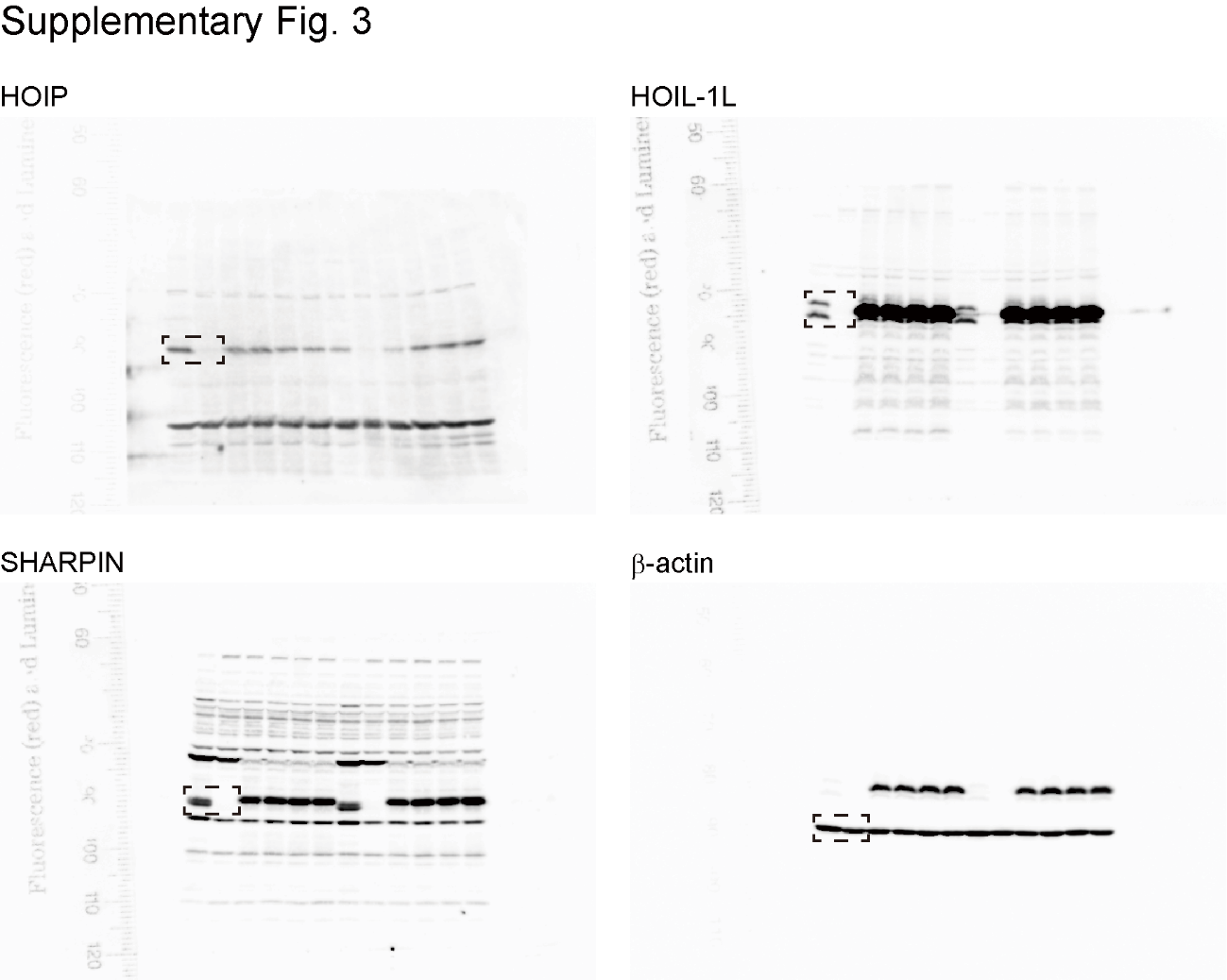


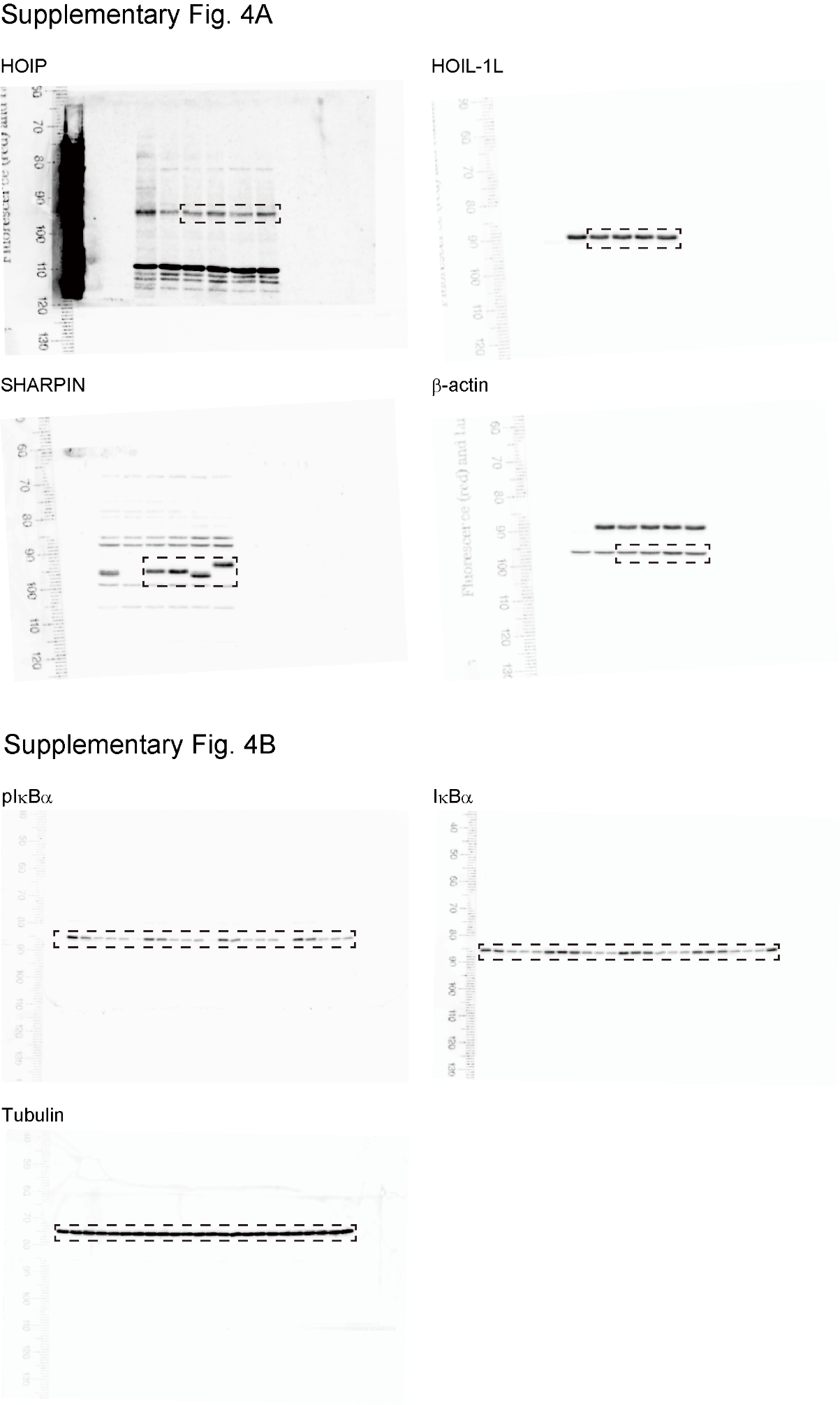


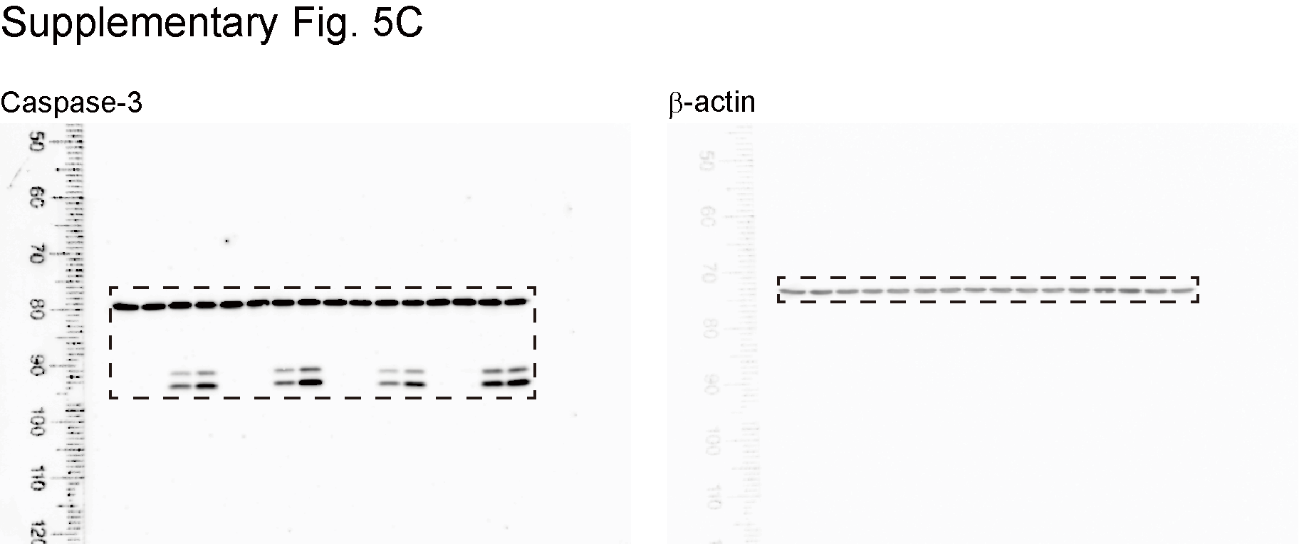


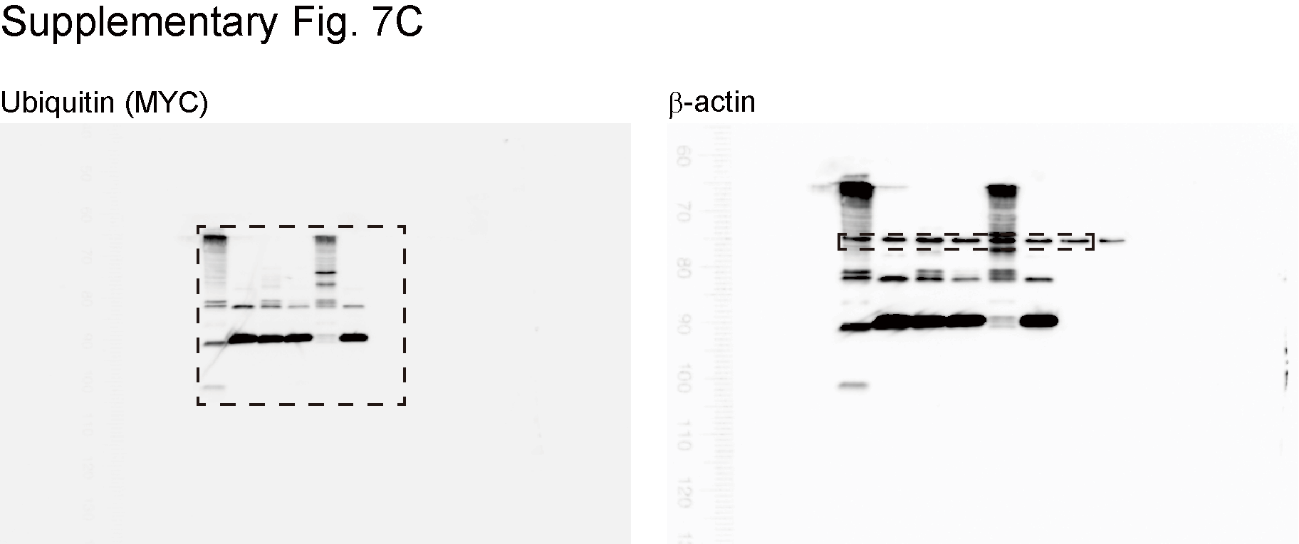


**Uncropped immunoblotting images**
